# Assessment of wood smoke induced pulmonary toxicity in normal- and chronic bronchitis-like bronchial and alveolar lung mucosa models at air–liquid interface

**DOI:** 10.1186/s12931-024-02686-5

**Published:** 2024-01-20

**Authors:** Swapna Upadhyay, Mizanur Rahman, Selina Rinaldi, Jeremy Koelmel, Elizabeth Z. Lin, Padukudru Anand Mahesh, Johannes Beckers, Gunnar Johanson, Krystal J. Godri Pollitt, Lena Palmberg, Martin Irmler, Koustav Ganguly

**Affiliations:** 1 Unit of Integrative Toxicology, Institute of Environmental Medicine (IMM), Karolinska Institutet, 171 77 Stockholm, Sweden; 2 Department of Environmental Health Sciences, Yale School of Public Health, Yale University, New Haven, CT USA; 3 Department of Respiratory Medicine, JSS Medical College, JSS Academy of Higher Education and Research, Mysore, 570015 India; 4 Institute of Experimental Genetics, Helmholtz Zentrum München, Deutsches Forschungszentrum Für Gesundheit Und Umwelt (GmbH), 85764 Neuherberg, Germany; 5 German Center for Diabetes Research (DZD E.V.), 85764 Neuherberg, Germany; 6 Chair of Experimental Genetics, Technical University of Munich, 85354 Freising, Germany

**Keywords:** Biomass, COPD, Asthma, Air pollution, Indoor, Household, HAP, SDG, Cilia, Ciliogenesis

## Abstract

**Background:**

Chronic obstructive pulmonary disease (COPD) has the highest increased risk due to household air pollution arising from biomass fuel burning. However, knowledge on COPD patho-mechanisms is mainly limited to tobacco smoke exposure. In this study, a repeated direct wood smoke (WS) exposure was performed using normal- (bro-ALI) and chronic bronchitis-like bronchial (bro-ALI-CB), and alveolar (alv-ALI) lung mucosa models at air–liquid interface (ALI) to assess broad toxicological end points.

**Methods:**

The bro-ALI and bro-ALI-CB models were developed using human primary bronchial epithelial cells and the alv-ALI model was developed using a representative type-II pneumocyte cell line. The lung models were exposed to WS (10 min/exposure; 5-exposures over 3-days; n = 6–7 independent experiments). Sham exposed samples served as control. WS composition was analyzed following passive sampling. Cytotoxicity, total cellular reactive oxygen species (ROS) and stress responsive NFkB were assessed by flow cytometry. WS exposure induced changes in gene expression were evaluated by RNA-seq (p ≤ 0.01) followed by pathway enrichment analysis. Secreted levels of proinflammatory cytokines were assessed in the basal media. Non-parametric statistical analysis was performed.

**Results:**

147 unique compounds were annotated in WS of which 42 compounds have inhalation toxicity (9 very high). WS exposure resulted in significantly increased ROS in bro-ALI (11.2%) and bro-ALI-CB (25.7%) along with correspondingly increased NFkB levels (bro-ALI: 35.6%; bro-ALI-CB: 18.1%). A total of 1262 (817-up and 445-down), 329 (141-up and 188-down), and 102 (33-up and 69-down) genes were differentially regulated in the WS-exposed bro-ALI, bro-ALI-CB, and alv-ALI models respectively. The enriched pathways included the terms acute phase response, mitochondrial dysfunction, inflammation, oxidative stress, NFkB, ROS, xenobiotic metabolism of AHR, and chronic respiratory disorder. The enrichment of the ‘cilium’ related genes was predominant in the WS-exposed bro-ALI (180-up and 7-down). The pathways primary ciliary dyskinesia, ciliopathy, and ciliary movement were enriched in both WS-exposed bro-ALI and bro-ALI-CB. Interleukin-6 and tumor necrosis factor-α were reduced (p < 0.05) in WS-exposed bro-ALI and bro-ALI-CB.

**Conclusion:**

Findings of this study indicate differential response to WS-exposure in different lung regions and in chronic bronchitis, a condition commonly associated with COPD. Further, the data suggests ciliopathy as a candidate pathway in relation to WS-exposure.

**Supplementary Information:**

The online version contains supplementary material available at 10.1186/s12931-024-02686-5.

## Introduction

Despite the significant progress made during the last decade towards achieving “Energy for All”, it is estimated that nearly 2 billion people will still lack access to clean cooking fuel by 2030 according to the 2023 “Energy Progress Report” [1]. Domestic activities such as cooking using open fires or traditional stoves, heating, and lighting results in household air pollution (HAP) [2, 3]. HAP is measured indoors and is predominant in the poor regions of low-middle income countries where access to electricity and clean cooking fuel is limited [2, 3]. Several other studies have also conveyed this understanding [4, 5]. also An impact assessment (2020) on HAP estimated 1.8 million (95% confidence interval/CI; 1.1–2.7) deaths and 60.8 million (34.6–93.3) disability-adjusted life-years globally in 2017 that is mainly borne by low-middle income countries [6]. Respiratory diseases accounted for 38% of all deaths (0.7 million; 0.4–1.0) and 75% of all disability-adjusted life-years (45.7 million; 26.8–68.8) attributable to HAP exposure in 2017 [6, 7]. Pooled relative risk (95% CI) assessment showed chronic obstructive pulmonary disease (COPD) to have the highest increased risk (1.70; 1.47–1.97) due to HAP exposure among all the adverse outcomes investigated [6, 7]. The use of efficient and cleaner fuel is directly linked to the income level of families as described in the energy ladder [8, 9]. Thus, women in poor rural areas of low-middle income countries spend on average four hours for cooking using biomass fuel. Women and children in these families therefore suffer the most from HAP [3, 6, 10, 11]. It has been reported that women exposed to HAP have defective phagocytosis and harbor potentially pathogenic bacteria with decline in lung function [12, 13]. Based on limited information, biomass smoke induced COPD exhibits greater bronchial involvement, less emphysema, more frequent hypoxia, and increased gas trapping compared to tobacco smoke induced COPD [11, 14–16]. This indicates exposure specific patho-mechanisms in biomass smoke induced COPD and tobacco smoke induced COPD. However, COPD in general, and exposure studies using in vitro and in vivo model systems to understand the molecular mechanisms of COPD has been mainly investigated in relation to tobacco smoke. Studies on biomass smoke induced COPD as well as exposure studies using biomass smoke are limited and the few available are mainly restricted to wood smoke extracted particulate matter (PM_2.5_) [17–23].

Smoke from combustion processes is a complex mixture. The material burnt and its moisture content, the temperature of the fire, and the aging of the smoke are some of the important factors contributing to the composition of smoke which in turn can influence toxicity [11, 24, 25]. The range of highly polluting fuels used for cooking and heating in poor and rural households of low-middle income countries includes wood, coal, crop waste, animal dung, charcoal, and kerosene which are often collectively referred to as biomass fuel [6]. The sources of fuel vary considerably based on climate, geography, and main occupation of the relevant population which in turn results in variation in the composition of biomass smoke [3]. Biomass smoke contains ultrafine, fine, and coarse particulate matter, carbon monoxide (CO), carbon dioxide (CO_2_), oxides of nitrogen and sulfur, polycyclic aromatic hydrocarbons (PAHs), aldehydes, free radicals, heavy metals, and chlorinated organic compounds [11, 26]. Furthermore, hundreds of volatile and semi-volatile organic compounds (VOCs) are released with various toxicities, although these are often uncharacterized in studies [27, 28]. Therefore, assessment of only the effects of biomass smoke derived particulate matter (such as PM_2.5_) toxicity may be inadequate to understand the broad spectrum of the toxicological response.

In this study we therefore aimed to assess the molecular toxicological response of wood smoke (representative biomass smoke) using our established physiologically relevant normal bronchial (bro), chronic bronchitis-like bronchial (CB), and alveolar (alv) lung mucosa models developed at air–liquid interface (ALI) following repeated exposures [29–36]. The broad toxicological endpoints identified for assessment were i) oxidative stress, ii) barrier function, iii) altered transcript expression, and iv) secretion of pro-inflammatory cytokines along with characterization of wood smoke composition. Pathway enrichment analysis was performed to identify biological mechanisms that may drive wood smoke mediated toxicity in different lung models. The normal bronchial mucosa models (bro-ALI) and chronic bronchitis-like bronchial (bro-ALI-CB) models were developed using human primary bronchial epithelial cells (PBEC) and the alveolar models (alv-ALI) was developed using representative human type II pneumocytes. Our study establishes a repeated wood smoke exposure system to assess in vitro the molecular toxicity in two different lung regions (bronchial and alveolar) as well as in a predisposed condition (chronic bronchitis) that is commonly associated with COPD.

## Materials and methods

**Fig. 1:**
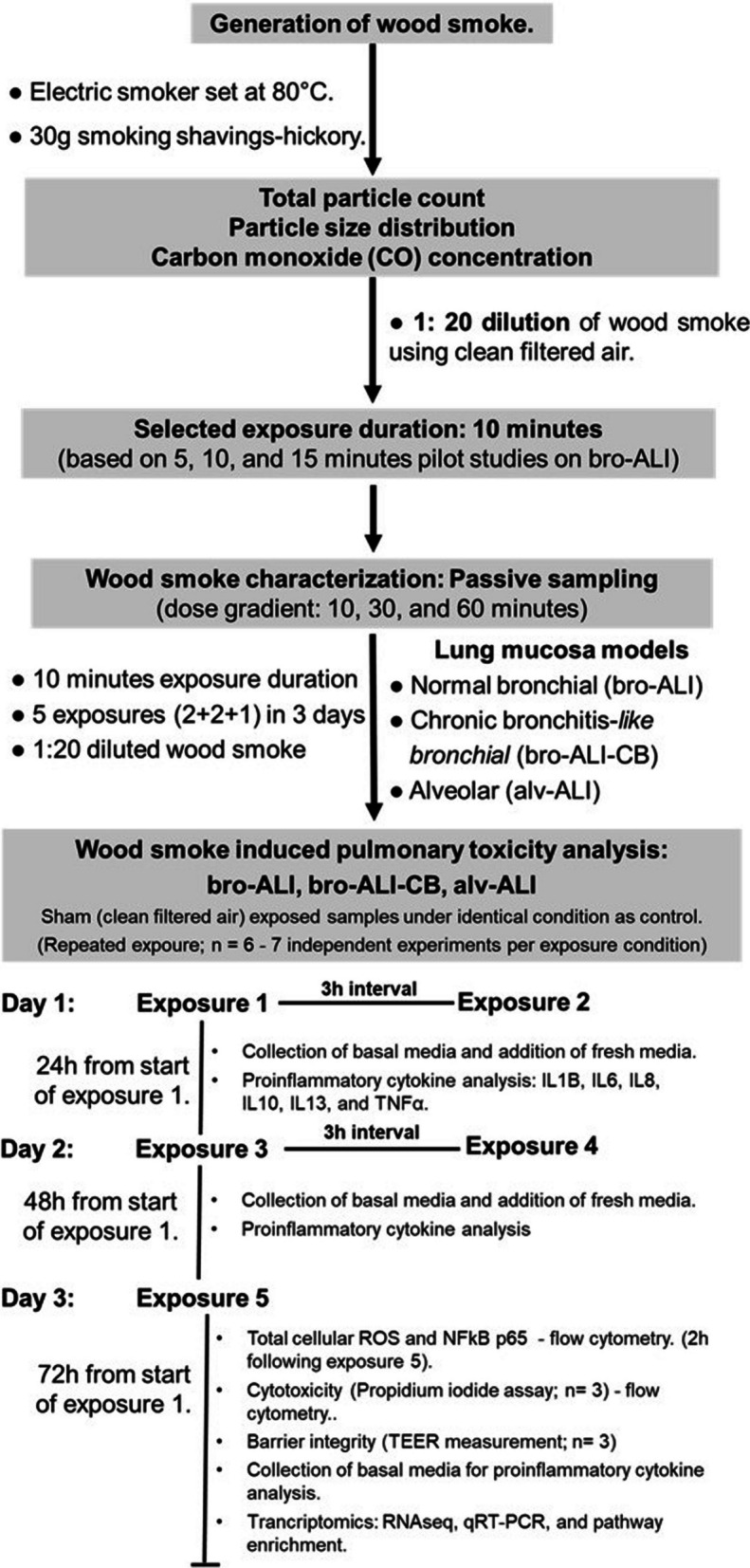
Schematic presentation of the overall experimental design outlining the wood smoke exposure regimen and analysis endpoints. ALI: air–liquid interface; alv-ALI: alveolar mucosa model at ALI; bro-ALI: normal bronchial mucosa model at ALI; bro-ALI-CB: chronic bronchitis-*like* bronchial mucosa model at ALI; CO: carbon monoxide; CB: chronic bronchitis; h: hours; H441: NCI-H441 (ATCC HTB-174) cell line; IQR: inter quartile range; IL: interleukin; LDH: lactate dehydrogenase; NFkB: nuclear factor kappa-light-chain-enhancer of activated B cells; PBEC: human primary bronchial epithelial cells; qRT-PCR: quantitative real time polymerase chain reaction; ROS: reactive oxygen species; SOD3: Superoxide dismutase 3, extracellular; TNF: tumor necrosis factor; TEER: transepithelial electrical resistance

An overall schematic presentation of the experimental design is shown in Fig. 1 and is illustrated in this section.

### Wood smoke generation

Pre-weighed 30 g of smoking shavings-hickory (MUSTANG; #246809; Kangasala, Finland) were used to generate wood smoke using a thermostat controlled electric smoker [MUSTANG; OGA339 #240429, Kangasala, Finland]. The temperature was set at 80 °C for smoke generation based on optimized protocol. The electric smoker was used according to the manufacturer’s instruction. Particle number concentration, size distribution, and CO concentration were measured in the smoke. CO concentration was used to decide the dilution of the wood smoke for exposure experiments to match realistic levels (~ 40 ppm) during cooking periods [37]. A defined weight of wood pellet heated at a regulated temperature provided a standardized smoke generation condition for exposure experiments.

### Particle number concentration and size distribution

A portable laser spectrometer [model Mini-LAS 11R; GRIMM, Aerosol Technik GmbH and Co. KG, Ainring, Germany) was used for measuring the particle number concentration (counts per cm^3^) and particle size distribution (instrument range 0.25–32 µm) of the wood smoke (n = 8 independent measurements) as previously described [34].

### CO concentration

CO concentration was measured using calibrated EasyLog data loggers with range 3–1000 ppm and − 10 to + 40 °C operating temperature (#EL-USB-CO, Lascar Electronics, Wiltshire, United Kingdom). The data loggers were placed inside the 3L desiccators used for exposure studies (n = 3 independent measurements).

### Wood smoke exposure system

**Fig. 2:**
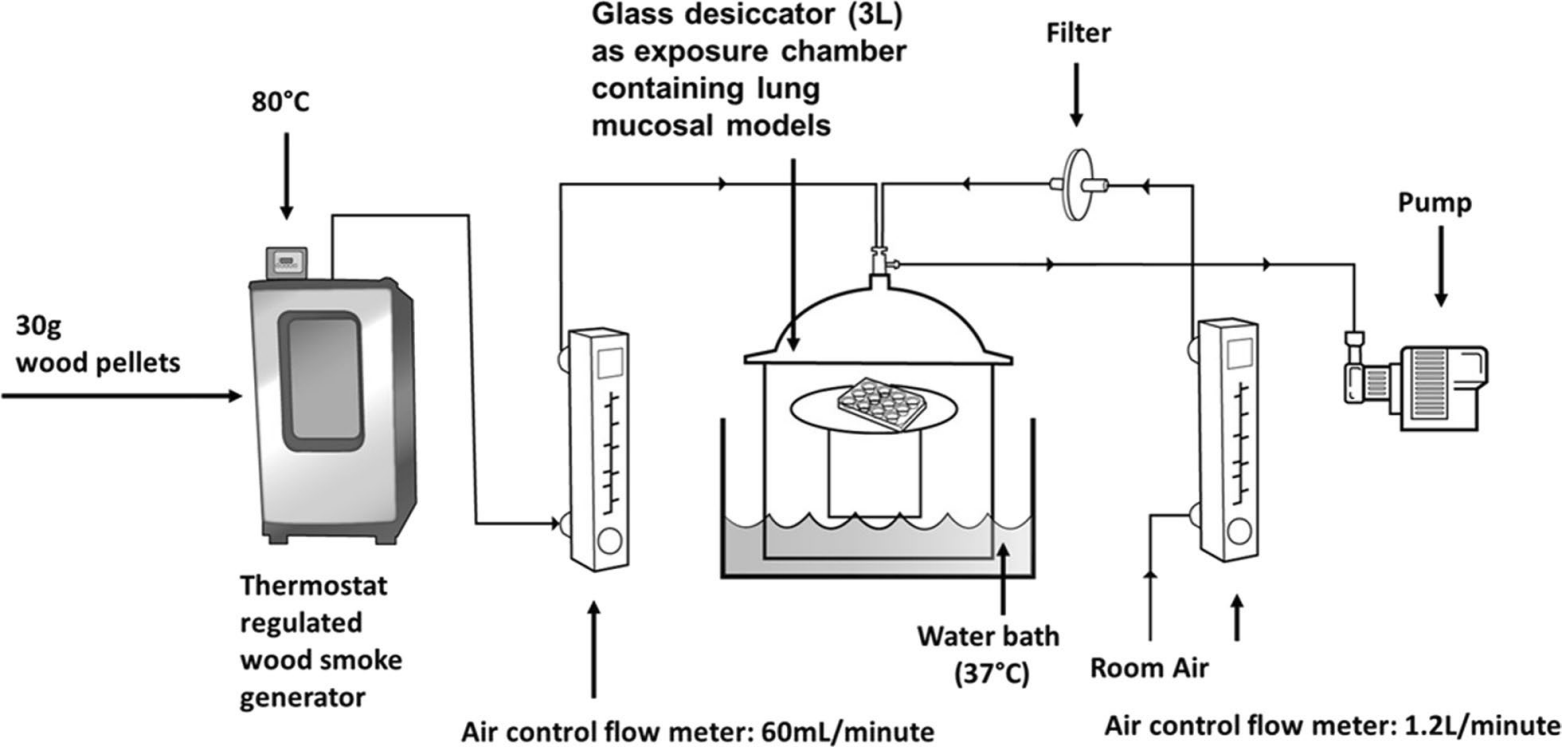
Schematic representation of the wood smoke exposure set up for normal bronchial (bro-ALI), chronic bronchitis-*like* bronchial (bro-ALI-CB), and alveolar (alv-ALI) lung mucosa models. The wood smoke was diluted (1:20) using filtered clean air. The authors sincerely acknowledge the assistance of Ann-Katrin Sjödén in preparing the figure

A schematic representation of the wood smoke exposure set up is provided in Fig. 2. The wood smoke was diluted in the ratio 1:20 with filtered clean air. The exposure duration of 10 min (min) was decided based on the findings of a pilot dose response experiment (5,10, and 15 min exposure duration) assessing cytotoxicity (lactate dehydrogenase assay in basal media) and transcript expression of tumor necrosis factor (*TNF;* proinflammatory marker) and *superoxide dismutase 3, extracellular* (*SOD3*; oxidative stress marker) (Additional file 1: Figure S1). The medium exposure dose was then chosen accordingly. None of the doses were cytotoxic.

The bro-ALI, bro-ALI-CB, and alv-ALI models were developed in transwell inserts in 12-well plates as previously described [29, 30, 34]. After change of cell medium, the plates were placed in a 3L desiccator glass jar maintained at 37 °C and 60% humidity and allowed to equilibrate for 5 min. The volume of the desiccator represents the functional residual capacity of a healthy adult human lung [38]. As the temperature of the electric smoker reached 80 °C, the pump was switched on to draw wood smoke at a flow rate of 60 ml per min along with filtered air at a flow rate of 1.2 L per min into the desiccator to obtain the 1:20 dilution.

The lung models were exposed to wood smoke or filtered clean air (Sham) for 10 min, where after they were transferred to a cell incubator (37 °C, 60% humidity and 5% CO_2_) for 3 h (h) until next exposure session on the same day (Fig. 1). Two exposure sessions were performed on days 1 and 2; and one exposure on day 3 (5 exposures in total; Fig. 1). Following completion of repeated exposures each day, the lung models were incubated for 24 h (since start of exposure on previous day). Cell culture medium was collected (and stored at − 80 °C) and replaced with fresh ALI medium 24 h after the first exposure each day and prior to the start of the exposure on the following day. Cell inserts were collected at the completion of 72 h since start of first exposure on day 1. The exposure regime did not induce cytotoxicity in any of bro-ALI, bro-ALI-CB, or alv-ALI models and therefore further molecular analysis was carried out. Cytotoxicity was assessed by lactate dehydrogenase (LDH; cat# 88953; Thermo Fisher scientific) for the pilot studies and propidium iodide (PI) staining (cat#556463; BD bioscience) for the repeated exposure studies. Sham exposed samples under identical conditions served as control.

All wood smoke exposed samples were compared to their corresponding sham exposed samples (n = 6–7 independent experiments per exposure condition; n = 3 independent experiments per exposure condition for cytotoxicity assay and barrier function assessment). The replicates were randomly distributed in the plates and experiments performed on different days were used for bro-ALI (developed from one donor; different vials), bro-ALI-CB (developed from the same donor as bro-ALI; different vials), and alv-ALI (developed from different cell vials) for the different assays.

### Characterization of wood smoke

To characterize the composition of wood smoke used for exposure, we performed a dose gradient analysis (10 min, 30 min, and 60 min).

*Sample introduction and* gas chromatography high resolution mass spectrometer* (GC-HRMS) acquisition:* Sample acquisition has previously been described [39]. Briefly, samples were placed in autosampler tubes, and a thermal desorption unit (Gerstel, Linthicum, MD, USA) was used to extract analytes, following which these analytes were cryo-focused to − 90 ºC on a 2 mm glass wool deactivated liner, in a cooled injection system (Gerstel, Linthicum, MD, USA), prior to transfer to the GC-HRMS. A 5% diphenyl 95% dimethyl slightly polar stationary phase was chosen for gas chromatography (TG-5SILMS, 30 m × 0.25 mm × 0.25 μm; ThermoFisher, Waltham, MA, USA) with a temperature gradient of 7 °C/min ramping from 70 °C to 300 °C. Samples were analyzed in electron impact (EI) ionization mode, with a scan range from *m/z* 53.4–800 at an acquisition rate of 4 Hz at 30,000 resolution on a Q-Exactive Orbitrap mass spectrometer (ThermoFisher, Waltham, MA, USA).

*Deconvolution, identification, and filtering:* Suspect screening has previously been described, and consisted of deconvolution and database searching. National Institute of Standards and Technology 2017 and GC orbitrap specific contaminant (2017) and polychlorinated biphenyls (PCB) EI spectral libraries were queried, screening over 250,000 unique compounds. Alkanes were used to calculate an alkane retention index, and only those with closely matching retention indices were retained, limiting the scope of the library to those with retention index information for similar columns. Annotations were filtered using the following filters: retention index total maximum deviation (experimental versus library): 50, and percent deviation: 1.5%; reverse search index greater than 600, reverse high-resolution filter greater than 75, and total score greater than 75. This filtering scheme has been validated as providing a high level of true positives, although it increases false negatives [39–42]. Furthermore, to reduce redundant, artifact, and background chemical features, blank feature filtering [43] and removal of duplicate compound identifications was performed. This scheme is based on an actionable annotation scoring framework recently published [41]. Chemicals which did not follow the observed increase across increased time of measurement, or which had low average total scores across all samples, were flagged.

*Chemical physical properties and toxicity:* Chemical and physical properties of molecules [44, 45], mammalian acute toxicity (rat oral LD_50_, 24 h) [46], and inhalation toxicity, were predicted or compiled. All predicted toxicity and physical properties were calculated using the Environmental Protection Agency (EPA) Chemical Dashboard [47] or for inhalation toxicity, the Hazard Comparison Dashboard was used to compile literature. Toxicity estimation software tool [45] and OPEn structure–activity/property Relationship App [44] models were used for calculating physical properties.

### Lung mucosal models at ALI

#### Normal- and chronic bronchitis-like bronchial mucosa models

The bro-ALI and bro-ALI-CB models were developed using PBEC harvested from macroscopically normal bronchial tissue obtained from one donor in connection with lobectomy following written and informed consent as described in previous studies [29–36]. The protocol was approved by the Swedish Ethical Review Authority (Institutional ethic committee reference number: 99–357; approved on 10th January 2000). All methods were performed in accordance with the relevant guidelines and regulations. The bro-ALI and bro-ALI-CB models were developed according to the procedure described previously [29, 30]. Interleukin 13 (IL13; 1 ng/ml) was added to the basal medium of each insert from the 1st day of PBEC in ALI condition to develop the bro-ALI-CB models. The detailed protocol and cellular differentiation (club cells, goblet cells, basal cells, ciliated cells, etc.) of the PBEC-ALI and PBEC-ALI-CB model development have been also described in detail previously [29, 30]. These models have been used in several other studies [29–36]. In general, lung mucosal models cultured at ALI have been widely employed in various studies as a model to study respiratory biology and diseases [48–53].

#### Alveolar model

The alv-ALI model was developed using NCI-H441 (ATCC HTB-174; derived from the pericardial fluid of a patient with papillary adenocarcinoma of the lung) cell line. The detailed protocol and model characteristics have been described in detail previously [34] and used in several recent studies [34–36]. The NCI-H441 cells, representative of human type II pneumocytes, express constitutively the mRNA and protein of the major surfactant apo-protein. NCI-H441 cells were co-cultured with HULEC-5a (ATCC CRL-3244) representative of human lung microvascular endothelial cells for this purpose. The alv-ALI model characterization included light microscopy, confocal microscopy, transmission electron microscopy, and transepithelial electrical resistance measurement that has been detailed previously [34]. Morphological characterization of the alv-ALI model demonstrated the presence of tight junction protein 1, lamellar bodies, surfactant protein C, microvilli, lipid bodies, desmosome, and tight junctions [34].

### Assessment of wood smoke related pulmonary response.

#### Cytotoxicity assessment

The membrane integrity-based cell viability assay, the PI assay, was used for cytotoxicity assessment of bro-ALI, bro-ALI-CB, and alv-ALI (n = 3 independent experiments per exposure condition) models following 72 h after the start of first exposure (i.e. at the completion of the entire exposure regime or exposure 5). Propidium iodide (BD bioscience, San Jose, CA, US, catalog # 556463) staining was used according to manufacturer’s instruction and as described previously [34–36]. The PI assay was performed using flow cytometry (BD LSRFortessa cell analyzer, BD bioscience, San Jose, CA, US). The flow cytometric data was analyzed using FlowJo software-7.6.1 (BD bioscience, San Jose, CA, US). Data are presented as percentage positive PI cells and interquartile ranges (IQR).

Colorimetric lactate dehydrogenase (LDH; Thermo Fisher scientific Rockford, lL, US, catalog # 88953) assay was used for assessing cytotoxicity only in the pilot experiments for optimization of the exposure protocol (Additional file 1: Figure S1). The LDH assay was measured using BioTek 800 TS absorbance reader (Santa Clara, CA, US) and absorbance was measured at 450 nm. The same instruments and software were used for all subsequent enzyme linked immune sorbent assays (ELISA) and flow cytometric analysis mentioned hereafter.

#### Total reactive oxygen species (ROS) and nuclear factor kappa-light-chain-enhancer of activated B cells (NFkB)

Total cellular ROS and NFkB were measured in bro-ALI, bro-ALI-CB, and alv-ALI (n = 6 independent experiments per exposure condition) models following 2 h post exposure 5 on day 3 (Fig. 1) using flow cytometry according to manufacturer’s instruction and described previously [34–36]. Briefly, cells were stained with CellROX green reagent (ThermoFisher Scientific, Rockford, lL, US, Catalog #: C10444) for 30 min in incubator. Following incubation, cells were washed, collected and ROS were measured. Expression of the stress responsive transcription factor NFkB was assessed following staining of the cells using NFkB p65 subunit kit (BD biosciences, San Jose, CA, US, catalog # 560335). Shortly, cells were stained with PE-Cy7 conjugated anti-NFkB p65 for 30 min, followed by washing, and measurement by flow cytometry. Mean fluorescent intensity (MFI) represents the level of total ROS and NFkB.

#### Barrier function

The barrier integrity of bro-ALI, bro-ALI-CB, and alv-ALI mucosal models following wood smoke exposure was assessed by measuring the transepithelial electrical resistance (TEER; Ω per cm^2^) with an EVOM Volt Ohm meter equipped with an EndOhm (World Precision Instruments, USA) as previously described [34]. Wood smoke exposed samples were compared to their corresponding sham exposed samples (n = 3 independent experiments per condition).

#### Transcriptomic analysis

Transcriptomic analysis was performed using the UPX 3′ RNA sequencing technology (RNAseq; Qiagen Genomic Services, Hilden, Germany) as previously described [31, 32]. To determine the differentially expressed genes following wood smoke exposure in bro-ALI, bro-ALI-CB, and alv-ALI (n = 6–7 independent experiments per exposure condition), cells were collected in Qiagen RLT buffer 24 h post exposure (Qiagen, Hilden, Germany, catalog # 74104), snap frozen, and dispatched in dry ice to the service laboratory according to the service provider’s instructions. For alv-ALI, the apical layer containing type II pneumocytes were collected. A raw p value ≤ 0.01 was set to select the differentially expressed genes. Gene symbols were obtained from Ensembl (BioMart) and NCBI (Gene2ensembl; Homo_sapiens.gene_info.gz). Heatmaps showing the top 25 up-regulated and 25 down-regulated genes (by fold change) were generated in R [54]. Ensembl genes without gene symbol annotation were omitted from heatmaps. RNAseq data is deposited at the Gene Expression Omnibus database at NCBI [GSE236857; https://www.ncbi.nlm.nih.gov/geo/; accessed on 17th January 2024]. Real time quantitative polymerase chain reaction (qRT-PCR) was performed on selective genes (n = 6 independent experiments per exposure condition) according to previously described methodology [34]. Actin beta (*ACTB*) was used as the reference control. The genes were selected based on their roles in barrier function (tight junction proteins: *TJP1*, *3;* claudins: *CLDN 1, 3, 4*; cadherin related family members: *CDHR3, 4*; protocadherin gamma subfamily A, 5: *PCDHGA5*), and in the mucin production (mucin, cell surface associated *MUC1, 12, 16*; mucin, oligomeric mucus/gel-forming: *MUC5AC*, *MUC5B*) together with significant differential regulation as detected in the RNAseq analysis.

#### Pathway and enrichment analysis

For the biological interpretation of the differentially regulated genes, canonical pathway and upstream regulator analyses were performed using the QIAGEN’s Ingenuity Pathway Analysis software (IPA, QIAGEN Redwood City, https://www.qiagen.com/ingenuity, content version 70750971, Release Date 2021-10-22) as previously described [35, 36]. Significant terms were selected using Fisher’s Exact Test p-values (p ≤ 0.05 for pathways and p ≤ 0.01 for up-stream regulators), z-scores ≥ 2 indicate activation, and z-scores ≤ − 2 indicate inhibition.

#### Secreted cytokine concentration

Concentrations of pro-inflammatory cytokines IL1β, IL6, IL8, IL10, IL13, and TNFα were measured in the basal media of the bro-ALI, bro-ALI-CB, and alv-ALI (n = 6–7 independent experiments per exposure condition) collected following 24 h, 48 h and 72 h after the start of exposure 1 as described previously [35, 36]. IL8 was measured using ELISA (R & D Systems, Minneapolis, MN, US, Catalog # DY208) and the remaining cytokines were measured using the V-plex immunoassay platform of Meso Scale Discovery Inc (Rockville, MD, US) at the Clinical Biomarkers facility, Science for Life Laboratory, Uppsala University, Sweden. Basal media of the samples used for RNAseq analysis were used for protein secretion analysis.

### Statistics

The results (flow cytometry, TEER, protein concentration, ELISA) were expressed as medians and IQR (25th–75th percentiles). The qRT-PCR data is expressed as median fold changes. Bro-ALI, bro-ALI-CB, and alv-ALI models are well-differentiated tissue-like models, which contain multiple layers of cells including different cell types of unique distribution. Hence, in this study, every model (i.e. bro-ALI, bro-ALI-CB, and alv-ALI) are considered as a unique in vivo-like in vitro model with its own distribution of different cell types and the number of cells present might differ. Further, the experiments were carried out on different days. Therefore, differences between treatments were examined by non-parametric statistical analysis (Wilcoxon signed rank test or Friedman test followed by Wilcoxon signed rank test, as appropriate) using GraphPad Prism (9.3.1) software (LaJolla, CA, US) as in previous studies [34–36]. A p value < 0.05 was considered as significant. The statistical method used in the RNA sequencing and pathway analysis are described in the respective sections.

## Results

### CO concentration and particle number concentrations

The CO concentration of wood smoke was 770 ± 45 ppm (n = 3 independent measurements: mean ± standard error of mean). The particle number concentration of wood smoke was 473.3 ± 86.6 counts per cm^3^ (n = 8 independent measurements). The particle size distribution ranged between 0.25 and 2.5 µm with the three peak mode diameters around 0.25, 0.58, and 0.28 µm in descending order (Additional file 1: Figure S2).

### Wood smoke composition

**Fig. 3:**
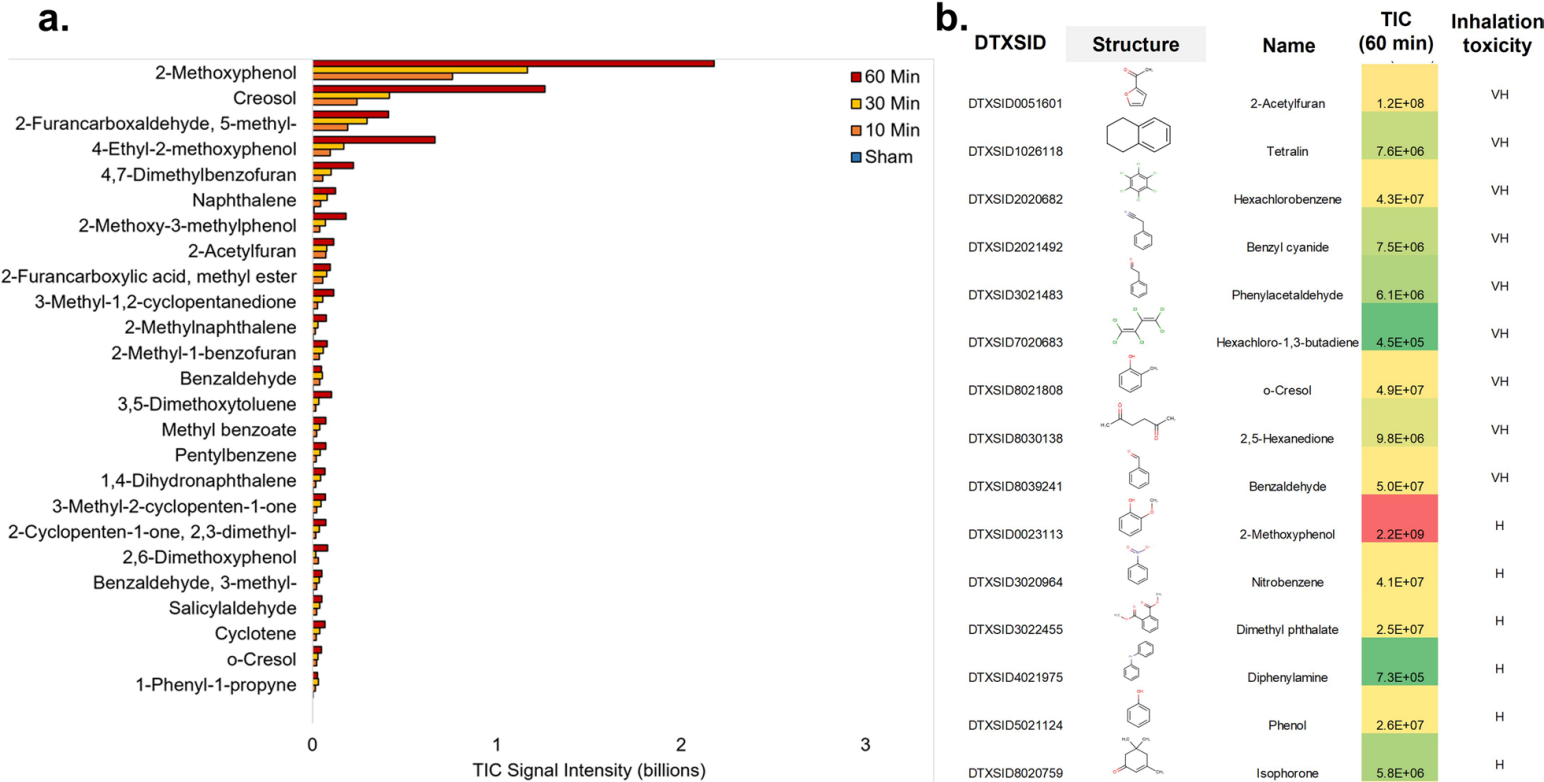
Characterization of wood smoke following 10, 30, and 60 min of air sampling. **a** Top 25 air pollutants identified in emissions released from wood smoke. Bar graphs show the measured total ion current (TIC) signal intensity for 10 to 60 min of air sampling as well as the sham. Chemicals which were flagged based on average score across samples and patterns across time of monitoring were not included. The color scale is based on the minimum, 50th percentile, and maximum of all 147 chemicals detected for each category. **b** Chemicals with Very High (VH) and High (H) Inhalation Toxicity based on the Environmental Protection Agency (EPA) Hazard Comparison Dashboard (HCD)

Wood smoke composition analysis tentatively identified 147 unique compounds (Additional file 1: B), many of which are known to be associated with combustion of wood, and 15% of which have also been found in cigarettes or cigarette smoke. A median R^2^ of 0.94, 25th percentile of 0.83, and 75th percentile of 0.97 was observed across all compounds (time versus compound signal intensities). For those with high correlations (> 0.9) an average increase of 515 ± 470% in measured signal abundances (total ion current) from 10 to 60 min of passive sampling was observed. The top 25 most abundant compounds based on total ion current and their structures are shown in Fig. 3a; with all structures sorted by abundance in Additional file 1: B. All of these chemicals are either furans, or contain a central benzene ring or 5-membered ring with various degrees of unsaturation. The remaining species alkyl carbon chains and/or aromatic rings containing oxygen constituents (e.g. ketones, aldehydes, and esters).

The compounds with the highest inhalation toxicity based on the hazard comparison dashboard are shown in Fig. 3b. The environmental protection agency hazard scores of low, moderate, high, or very high hazard are based on the DfE Alternatives Assessment Criteria for Hazard Evaluation. This criterion provides guidance for converting values from a variety of different sources and formats into the consistent low, moderate, high, or very high scores [55]. For inhalation toxicity only 42 of the 147 unique compounds had animal studies indicating mammalian inhalation toxicity linked to the hazard comparison dashboard. Of these 42 compounds, 21 chemicals had inconclusive evidence regarding inhalation toxicity. Of the remaining 21 chemicals, 9 were considered to have “Very High” inhalation toxicity; these chemicals were: 2-acetylfuran, tetralin, hexachlorobenzene, benzyl cyanide, phenylacetaldehyde, hexachloro-1,3-butadiene, o-cresol, 2,5-hexanedione, and benzaldehyde, and their structures are shown in Fig. 3b. It is important to note that some most toxic species (Fig. 3b) were also the most abundant (Fig. 3a) and hence these compounds are of specific concern: 2-acetylfuran, o-cresol, and benzaldehyde.

### Cytotoxicity

The repeated exposure regime used in this study was not cytotoxic (> 85% cell viability) in any of the lung mucosa models models (n = 3 independent experiments per exposure condition) as detected by PI staining (Additional file 1: Figure S3). In the case of wood smoke exposed bro-ALI-CB (9.5%; p = 0.0019) and alv-ALI (4.9%; p = 0013) but not for bro-ALI, significantly increased PI positive cells were observed compared to the corresponding sham exposed samples.

### Oxidative stress

**Fig. 4:**
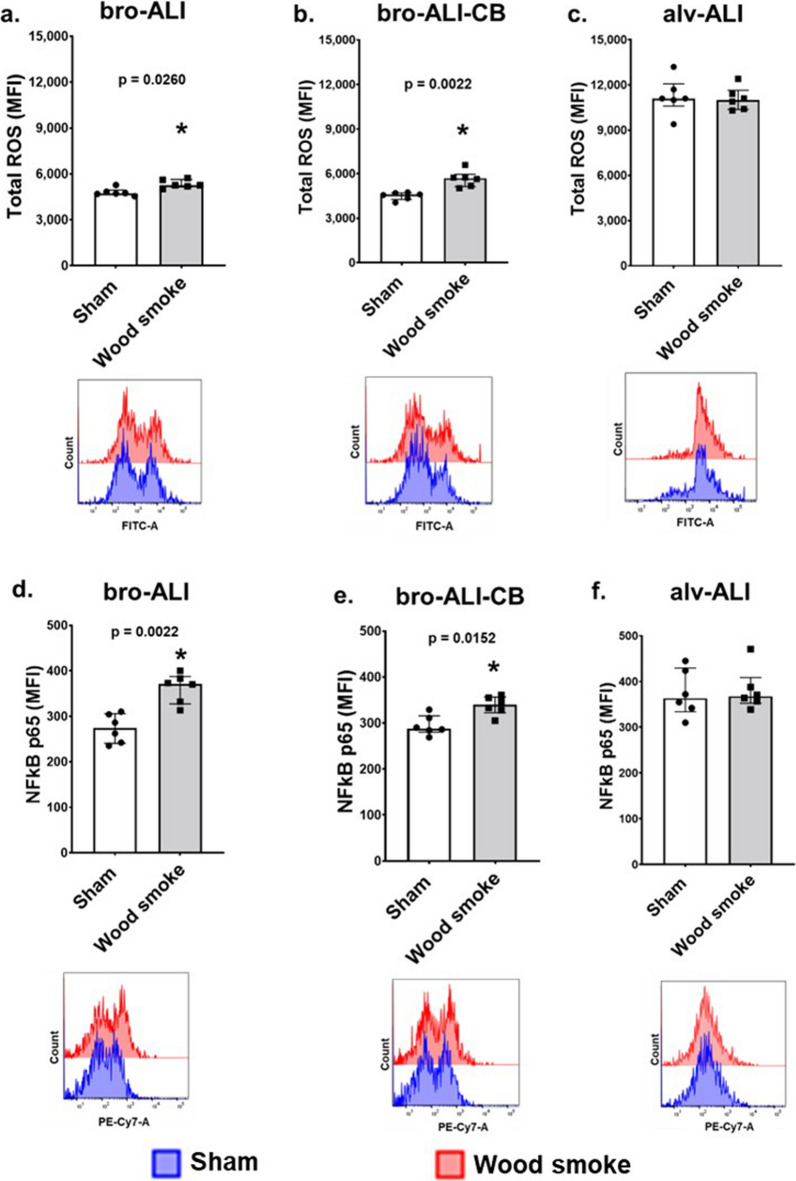
Assessment of oxidative stress response by measurement of **(a–c**) total cellular reactive oxygen species (ROS) and **(d–f)** expression of nuclear factor kappa-light-chain-enhancer of activated B cells (NFkB) p65 subunit by flow cytometry in sham exposed and wood smoke exposed bro-ALI, bro-ALI-CB, and alv-ALI models. Data are shown as medians and interquartile ranges. Representative histograms of flow cytometric assessment of ROS and NFkB for each condition are shown along with the respective bar graphs. n = 6 independent experiments per exposure condition; non-parametric statistical analysis (Wilcoxon signed rank test), *p < 0.05. ALI: air–liquid interface; alv-ALI: alveolar mucosa model at ALI; bro-ALI: normal bronchial mucosa model at ALI; bro-ALI-CB: chronic bronchitis-like bronchial lung mucosa model, FITC: Fluorescein, MFI: mean fluorescent intensity, PE-Cy7: PE-Cyanine7

Increased total cellular ROS was detected in bro-ALI (11.2%; p = 0.0260; Fig. 4a) and bro-ALI-CB (25.7%; p = 0.0022; Fig. 4b) but not in alv-ALI (Fig. 4c) following wood smoke exposure compared to corresponding sham (n = 6 independent experiments per exposure condition). Correspondingly, levels of NFkB p65 subunit were also increased in bro-ALI (35.6%; p = 0.0022; Fig. 4d) and bro-ALI-CB (18.1%; p = 0.0152; Fig. 4d) but not in the case of alv-ALI (Fig. 4f) following wood smoke exposure compared to sham (n = 6 independent experiments per exposure condition).

### Barrier function

The exposure regimen followed in this study did not cause any significant changes in the barrier integrity of wood smoke exposed bro-ALI, bro-ALI-CB, and alv-ALI compared to the corresponding sham as observed by TEER measurements (n = 3 independent experiments per exposure condition). [TEER (Ω per cm^2^; median, IQR); bro-ALI: sham: 277 (250; 292), wood smoke 292 (274.4; 306); bro-ALI-CB: sham: 225 (219; 225.5), wood smoke: 213 (198; 218.5); alv-ALI: sham: 269 (245.5; 279.5) vs wood smoke 286 (241; 296)].

### Transcriptomic alterations and proinflammatory cytokine secretion

#### Normal bronchial mucosa model (bro-ALI)

**Fig. 5:**
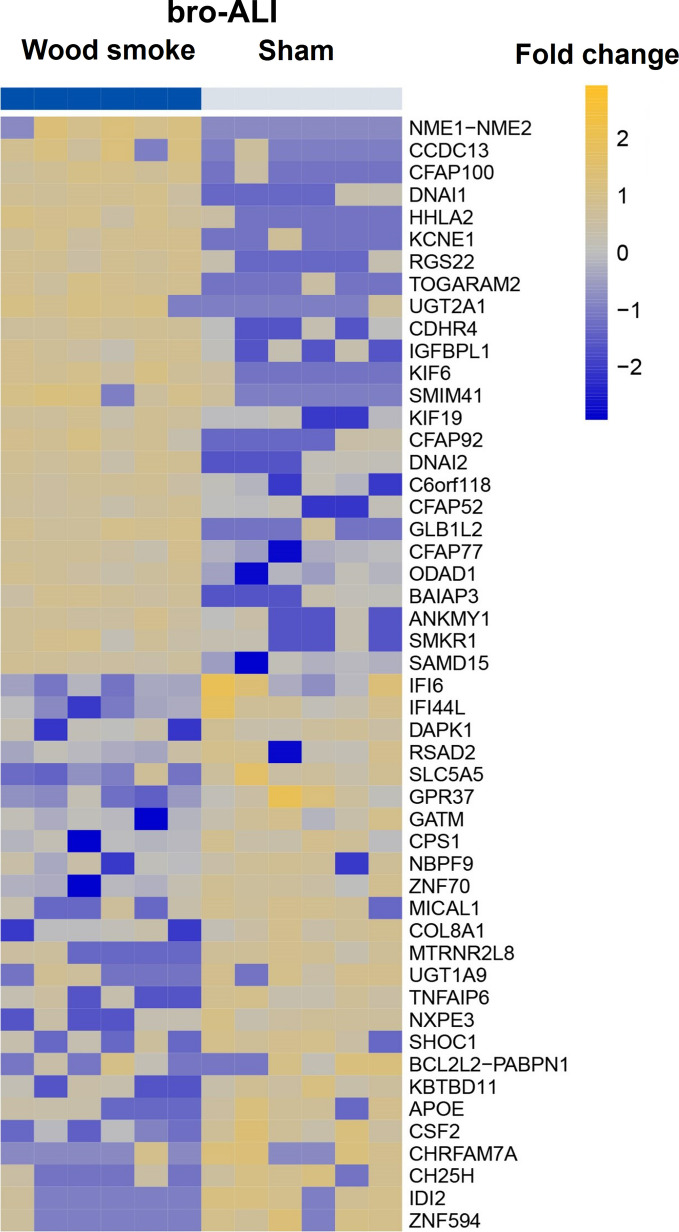
Heatmap of the top 25 up-regulated and 25 down-regulated genes in the bro-ALI model following repeated exposure to wood smoke. Significantly regulated genes (p ≤ 0.01) with the highest fold changes are shown and relative gene expression values are shown across samples (z-scales to mean expression per row). A complete list of the 1262 differentially regulated genes is provided in Additional file 1: table ST1. n = 6 independent experiments per exposure condition; bro-ALI: bronchial mucosa model at air–liquid interface, bro-ALI: normal bronchial mucosa model at air–liquid interface

**Fig. 6:**
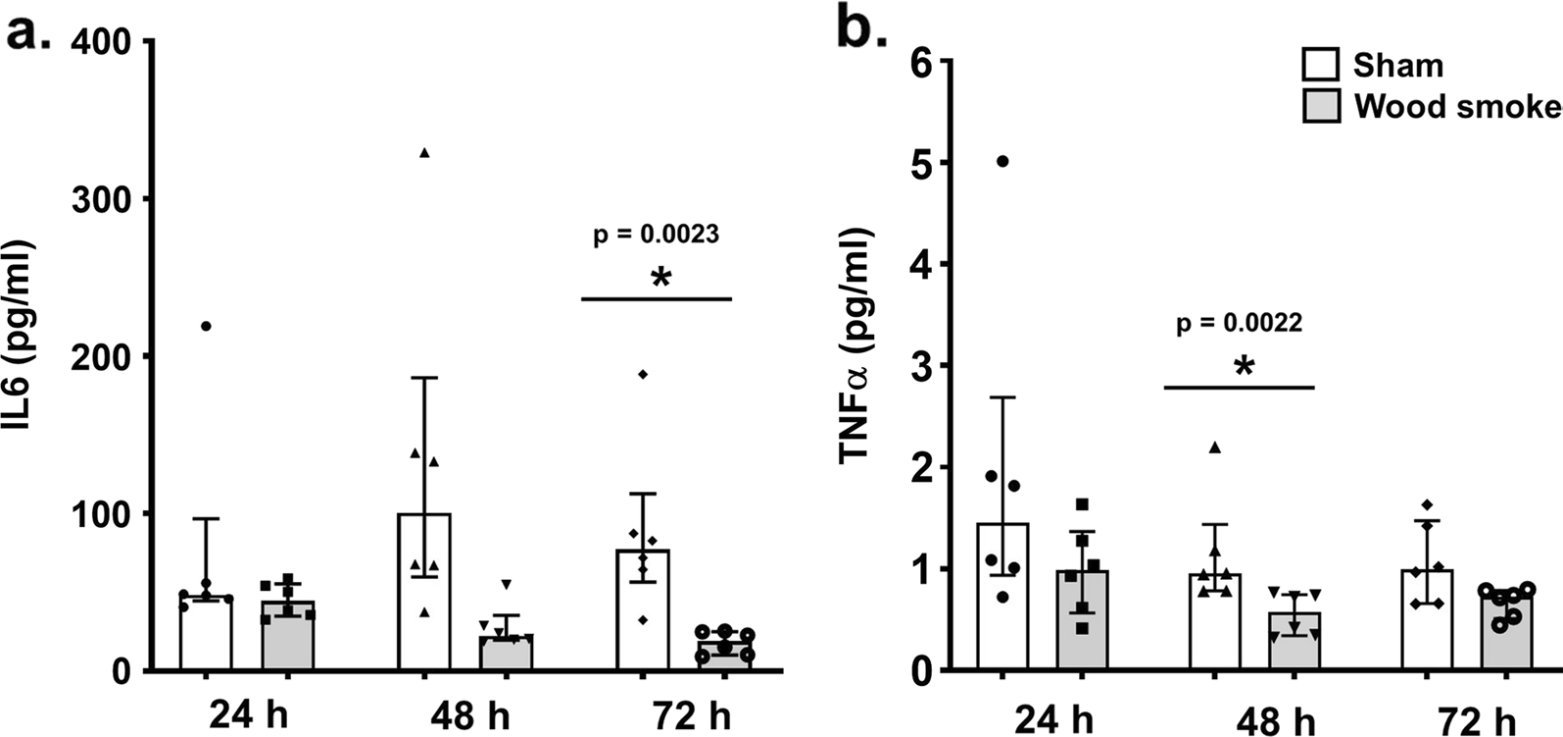
Concentration of secreted interleukin 6 (IL6) and tumor necrosis factor alpha (TNFα) in the basal media of sham exposed and wood smoke exposed bro-ALI model during the repeated exposure regime. The concentrations of cytokines have been measured at 24 h, 48 h and 72 h from the first exposure. Data are shown as medians and interquartile ranges. n = 6 independent experiments per exposure condition; non-parametric statistical analysis (Wilcoxon signed rank test); *Significance: p < 0.05. bro-ALI: normal bronchial mucosa model at air–liquid interface

A total of 1262 genes were differentially regulated (817 up-regulated and 445 down-regulated; n = 6 independent experiments per exposure condition; p ≤ 0.01) in the bro-ALI model following wood smoke exposure compared to sham (Additional file 1: table ST1). Figure 5 shows a heat map of the top 25 up-regulated and 25 down-regulated genes. Enrichment analysis using the 1262 differentially regulated genes identified 56 significantly enriched canonical pathways in the bro-ALI model (Additional file 1: Table ST2). On protein level, significantly reduced levels of the secreted proinflammatory cytokines IL6 (p = 0.0023; Fig. 6a) and TNFα (p = 0.0022; Fig. 6b) were detected at 72 h and 48 h respectively (since start of exposure 1) in the bro-ALI model following exposure to wood smoke (n = 6 independent experiments per exposure condition). None of the other examined cytokines was significantly altered by exposure to wood smoke in the bro-ALI model.

#### Chronic bronchitis-like bronchial mucosa model (bro-ALI-CB)

**Fig. 7:**
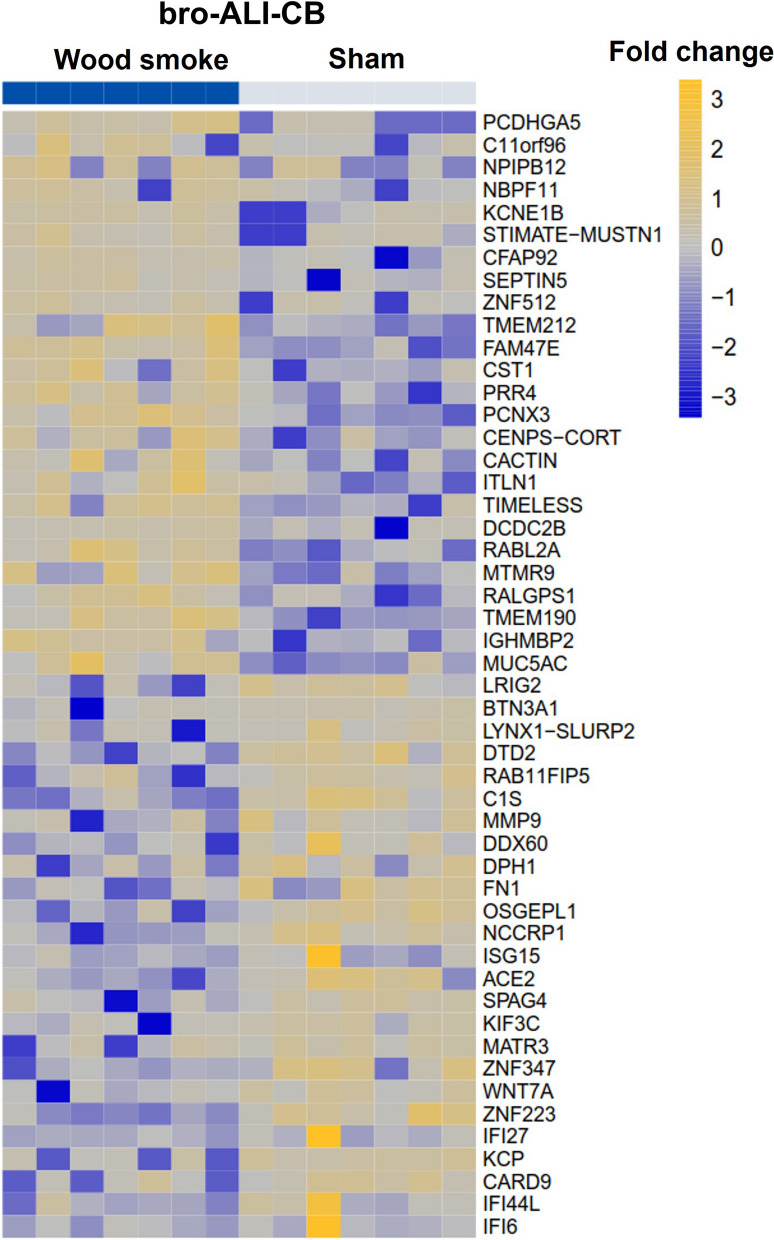
Heatmap of the top 25 up-regulated and 25 down-regulated genes in the bro-ALI-CB model following repeated exposure to wood smoke. Significantly regulated genes (p ≤ 0.01) with the highest fold changes are shown and relative gene expression values are shown across samples (z-scales to mean expression per row). A complete list of the 329 differentially regulated genes is provided in Additional file 1: table ST3. n = 7 independent experiments per exposure condition; bro-ALI: normal bronchial mucosa model at air–liquid interface; bro-ALI-CB: chronic bronchitis-like bronchial mucosa model at ALI

A total of 329 genes were differentially regulated (141 up-regulated and 188 down-regulated; n = 7 independent experiments per exposure condition; p ≤ 0.01) in the bro-ALI-CB model following wood smoke exposure compared to sham (Additional file 1: table ST3). Figure 7 shows a heat map of the top 25 up-regulated and 25 down-regulated genes. Enrichment analysis using the 329 differentially regulated genes identified 36 significantly enriched canonical pathways in the bro-ALI-CB model (Additional file 1: Table ST4).

**Fig. 8:**
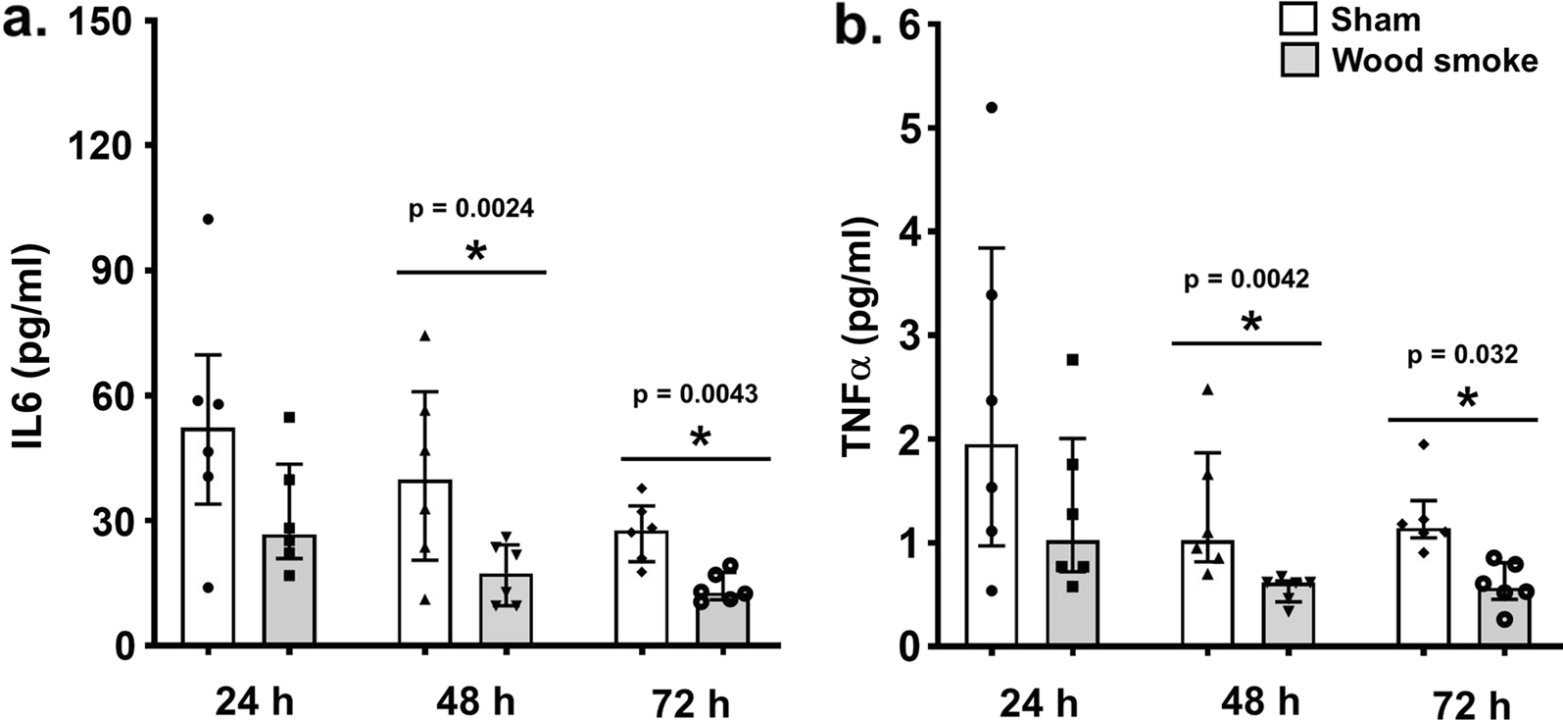
Concentration of secreted interleukin 6 (IL6) and tumor necrosis factor alpha (TNFα) in the basal media of sham exposed and wood smoke exposed bro-ALI-CB model during the repeated exposure regime. The concentrations of cytokines have been measured at 24 h, 48 h and 72 h from the first exposure. Data are shown as medians and interquartile ranges. n = 6 per exposure condition; non-parametric statistical analysis (Wilcoxon signed rank test); *Significance: p < 0.05. bro-ALI: normal bronchial mucosa model at air–liquid interface; bro-ALI-CB: chronic bronchitis-like bronchial mucosa model at ALI

Significantly reduced levels of the secreted proinflammatory cytokines IL6 (p = 0.0024 and 0.0043; Fig. 8a) and TNFα (p = 0.0042 and 0.032; Fig. 8b) were detected at both 48 h and 72 h (since start of exposure 1) in the bro-ALI-CB model following exposure to wood smoke (n = 7 independent experiments per exposure condition). None of the other examined cytokines was significantly altered by exposure to wood smoke in the bro-ALI-CB model.

#### Alveolar mucosa model (alv-ALI)

**Fig. 9:**
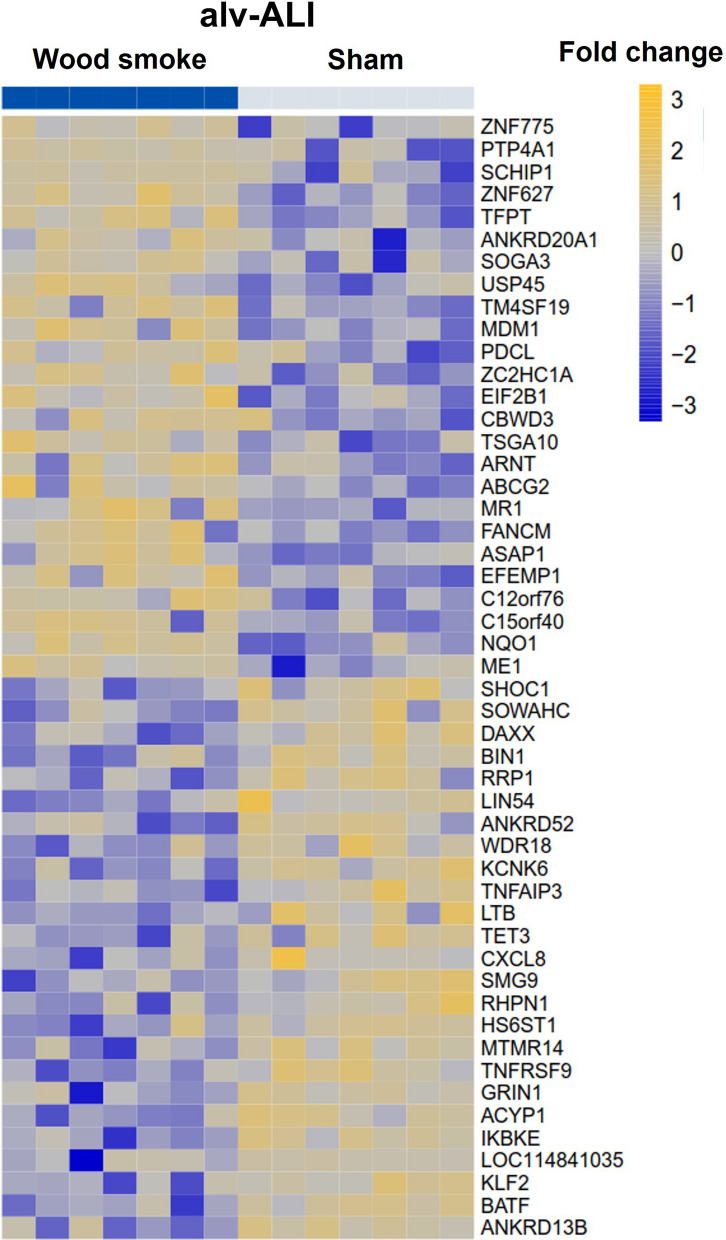
Heatmap of the top 25 up-regulated and 25 down-regulated genes in the alv-ALI model following repeated exposure to wood smoke. Significantly regulated genes (p ≤ 0.01) with the highest fold changes are shown and relative gene expression values are shown across samples (z-scales to mean expression per row). A complete list of the 102 differentially regulated genes is provided in Additional file 1: table ST5. n = 7 independent experiments per exposure condition; alv-ALI: alveolar mucosa model at air–liquid interface

A total of 102 genes were differentially regulated (33 up-regulated and 69 down-regulated; n = 7 independent experiments per exposure condition; p ≤ 0.01) in the alv-ALI model following wood smoke exposure compared to sham (Additional file 1: Table ST5). Figure 9 shows a heat map of the top 25 up-regulated and 25 down-regulated genes. Enrichment analysis using the 102 differentially regulated genes identified 108 significantly enriched canonical pathways in the alv-ALI model (Additional file 1: Table ST6). Secreted levels of none of the examined proinflammatory cytokines were significantly altered by exposure to wood smoke in the alv-ALI model.

#### Pathway enrichment analysis

**Fig. 10:**
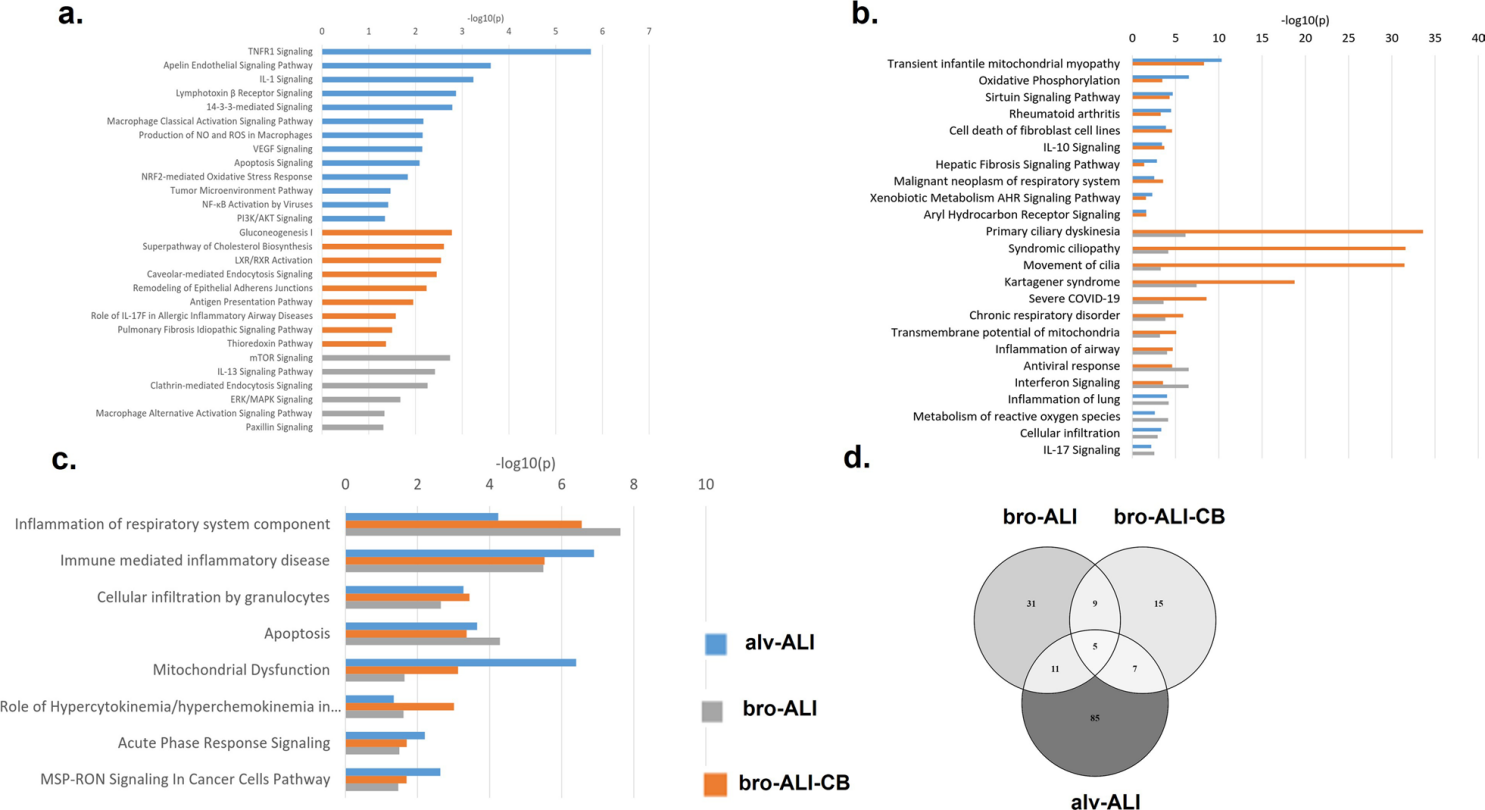
Graphical representation of enriched canonical pathways in normal bronchial (bro-ALI), chronic bronchitis-like bronchial (bro-ALI-CB), and alveolar (alv-ALI) lung mucosa models developed at air–liquid interface (ALI) following repeated wood smoke exposure compared to the corresponding sham. **a** Enriched pathways in either (i.e. not common pathways) bro-ALI or bro-ALI-CB or alv-ALI models. **b** Enriched pathways common between two lung models. **c** Pathways enriched among bro-ALI, bro-ALI-CB, and alv-ALI i.e. all the three lung models. **d** Graphical representation of the overlap of enriched pathways among bro-ALI, bro-ALI-CB, and alv-ALI models. The list of significantly differentially regulated genes used identified in the transcriptomic analysis was used as input (bro-ALI: 1262 genes; bro-ALI-CB: 329 genes; alv-ALI: 102 genes; Additional file 1: Tables ST1, ST3, and ST5). The canonical pathways listed here were identified using QIAGEN’s Ingenuity Pathway Analysis software (IPA®). Significant terms were selected using Fisher’s Exact Test p-values

**Fig. 11:**
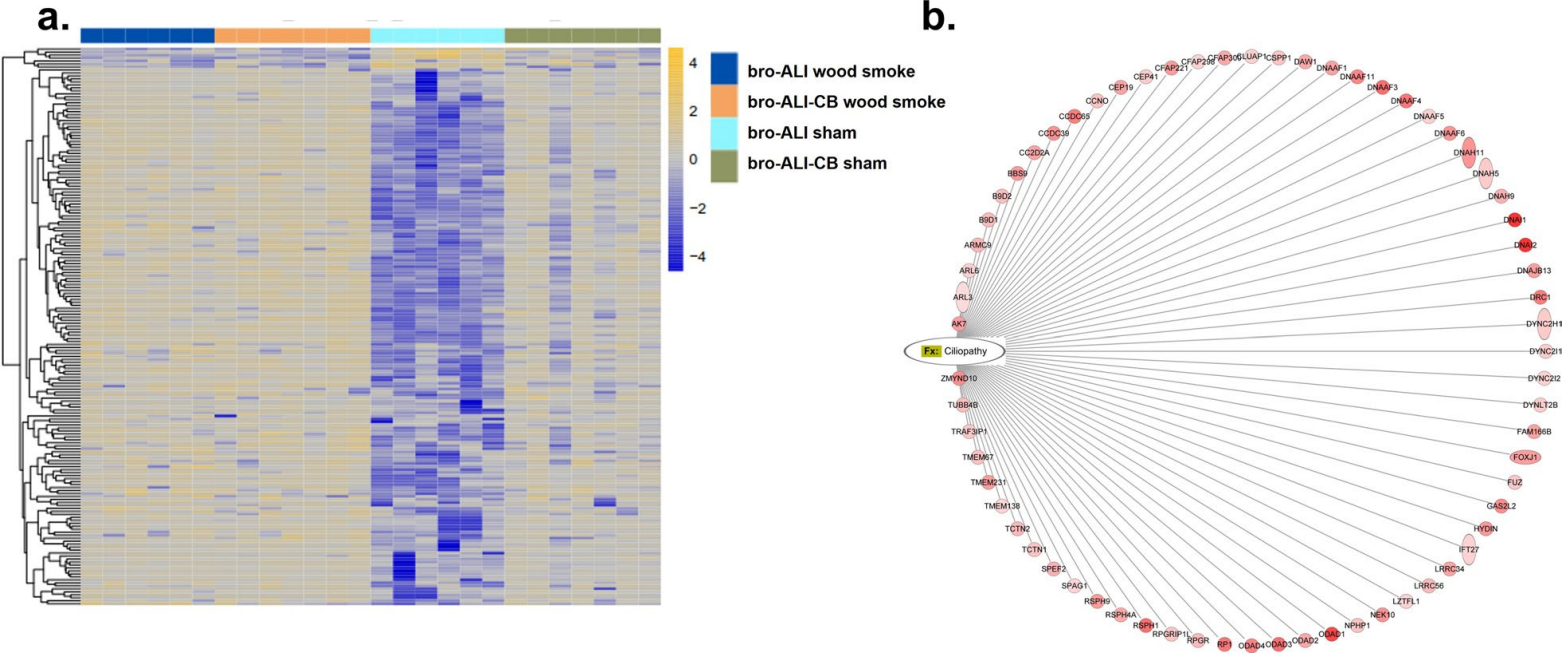
An overview of the regulation of cilia related genes (cilium; Gene ontology term: 0005929) in the normal bronchial (bro-ALI) and chronic bronchitis-like bronchial (bro-ALI-CB) lung mucosa models following wood smoke exposure compared to corresponding sham exposed samples. **a** 187 cilia related genes are differentially regulated in the bro-ALI (p ≤ 0.01; 1262 sets). **b** Ciliopathy related genes (p ≤ 0.01) are all up-regulated in bro-ALI. bro-ALI: normal bronchial mucosa model at air–liquid interface; bro-ALI-CB: chronic bronchitis-like bronchial mucosa model at ALI

Three different scenarios (Fig. 10) were obtained in the pathway enrichment analysis: (i) pathways unique to either bro-ALI or bro-ALI-CB or alv-ALI (Fig. 10a); (ii) pathways common to either bro-ALI and bro-ALI-CB, or bro-ALI and alv-ALI, or bro-ALI-CB and alv-ALI (Fig. 10b); and (iii) pathways common to bro-ALI, bro-ALI-CB, and alv-ALI (Fig. 10c). The overlap of the number of pathways between the different lung mucosa models is shown in Fig. 10d. Pathways enriched in all the three models included acute phase response signaling, mitochondrial dysfunction, macrophage stimulating protein (MSP)–Recepteur d'origine nantais (RON) signaling in cancer cells pathway, role of hyper-cytokinemia / hyper-chemokinemia in the pathogenesis of influenza, apoptosis, cellular infiltration by granulocytes, immune mediated inflammatory disease, and inflammation of respiratory system components. The pathways primary ciliary dyskinesia, syndromic ciliopathy, movement of cilia, and Kartagener syndrome are enriched in both bro-ALI and bro-ALI-CB models. The enrichment of the cilium related genes is particularly predominant in the bro-ALI model. Figure 11a shows the heat map of 187 cilia related genes (gene ontology terms: ‘Cilium’-GO 0005929 or ‘motile cilium’-GO0031514; 180 up-regulated and 7 down-regulated) out of the 1262 genes which were differentially regulated in the wood smoke exposed bro-ALI model compared to the corresponding sham (Additional file 1: Table ST7). In the case of bro-ALI-CB, 26 genes had altered expression levels (25 up-regulated, 1 down-regulated) out of the 187 genes belonging to the GO terms ‘Cilium’ or ‘motile cilium’ which were differentially regulated following wood smoke exposure compared to sham (p ≤ 0.01). All significantly regulated genes related to ciliopathy were up-regulated in the bro-ALI model (Figs. 11b).

#### Assessment of targeted genes by qRT-PCR

Expression of selected genes (n = 6 independent experiments per exposure condition; median fold change; p < 0.05) involved in barrier function (*TJP1*, *3; CLDN 1, 3, 4*; *CDHR3, 4*; *PCDHGA5*) and mucin secretion (*MUC1, 5AC, 5B, 12, 16*) assessed by qRT-PCR analysis (Additional file 1: table ST8) were consistent with the findings of RNAseq data (Additional file 1: Tables ST1, ST3, ST5). Among the barrier function genes screened by qRT-PCR, significantly increased expression of *CLDN3* (threefold), *CDHR3* (twofold), and *CDHR4* (sevenfold) was detected in wood smoke exposed bro-ALI whereas only *PCDHGA5* (sevenfold) was significantly increased in case of wood smoke exposed bro-ALI-CB compared to the corresponding sham controls (Additional file 1: Table ST8). Among the mucin related genes assessed by qRT-PCR, significantly increased expression of *MUC1* (1.5-fold), *MUC5AC* (2.5-fold), and *MUC5B* (fivefold) were detected in wood smoke exposed bro-ALI-CB whereas only *MUC12* (3.5-fold) was significantly increased in the wood smoke exposed bro-ALI model compared to the corresponding sham controls (Additional file 1: Table ST8).

## Discussion

In this study direct wood smoke exposure (repeated) was performed in physiologically relevant human normal and chronic bronchitis-like bronchial, and alveolar lung mucosa models at ALI. The experimental protocol reasonably mimicked a daily exposure scenario (e.g. twice for cooking two meals and CO concentration) in households using solid fuel for cooking. The bro-ALI and alv-ALI models represent the conducting and respiratory zones of the lung respectively and the bro-ALI-CB represents a predisposed condition (chronic bronchitis) commonly associated with COPD. Furthermore, exposure to biomass smoke is an established risk factor for chronic bronchitis among women in poor households using solid fuel for cooking [56]. Hence, we assessed the adverse effects of wood smoke exposure under three different physiological conditions (normal- and chronic bronchitis-like bronchial models, and alveolar models). Further, Ghosh et al., [57] demonstrated that 96% of the expressed genes show similar expression profiles between epithelial cells cultured at ALI and cells obtained from the freshly brushed nasal epithelia of the same patient. Such findings reinforce the relevance of lung mucosal models developed at ALI to assess the effects of inhaled toxicants as also discussed by others [52, 53].

It has been demonstrated that fuel type and combustion phase of biomass smoke influence toxicity [24, 25]. Therefore, we used a standardized condition for wood smoke generation by maintaining the temperature of the electric smoker and using a defined mass of hickory wood for experimental exposure studies. The CO concentration was used to dilute smoke matching realistic indoor CO levels reported from households using biomass fuel for cooking [37]. Our measurements of the particle size diameter of the wood smoke ranged from 0.25 µm to 2.5 µm, with the highest particle number concentration abundance between 0.25 and 0.58 µm. The lower range of the instrument used was 0.25 µm. Studies reported the average geometric mean diameter of wood smoke particles generated from birch, pine, and spruce emissions as 178 nm, 110 nm, and 91 nm respectively [23, 24]. Hence, both bronchial and alveolar lung mucosal models are relevant and useful to assess adverse effects at different levels of the respiratory tree. However, as discussed previously [34], it is challenging to translate the dosimetry from our in vitro exposure system to the lung in vivo. The direct exposure method used in this study using physiologically relevant human lung mucosal models are however more realistic than using wood smoke extracts and submerged cell culture systems.

As mentioned earlier, only a few studies have performed direct wood smoke exposure in vitro or in vivo. Thus, toxicological assessment of the effects of gaseous and particulate phase of the wood smoke is often lacking. The median R^2^ of 0.94, 25th percentile of 0.83, and 75th percentile of 0.97 observed across all the compounds (time versus compound signal intensities) for wood smoke characterization in this study shows that passive sampling can be used to measure a linear increase in exposure to wood smoke across time for most chemicals. The dominant species reflect biomass burning previously characterized by GC X GC mass spectrometry [58]. Polycyclic and cyclic aromatic hydrocarbons and oxygenated derivatives, oxygenated cyclic hydrocarbons, and furans, were previously characterized as dominant components of biomass burning [58]; these classifications cover the entire chemical space of the top 25 measurements in this study. In addition, specific components measured also overlapped between this study and the previous comprehensive study, including 1-alkyl phenol, benzofuran and methylated derivates, benzaldehyde, and methyl furans and furfurals [58]. Furthermore, multiple guaiacol and related derivatives, which are major components of coniferous species due to guaiacyl units predominantly forming the lignin in conifers, were measured at high levels both in this study and previously [58]. All of these detected chemicals, especially guaiacol and derivatives and furans, have high secondary organic aerosol formation potential [58], which signifies their important role in particulate matter formation which has a range of health hazards.

Of the combustion products annotated in our study with very high and high toxicity, 2-methoxyphenol [59, 60], phenol [61], 2-acetylfuran [62], tetralin [63], hexachlorobenzene [64], o-cresol [65], and benzaldehyde [66] have all been shown previously to be non-negligible combustion products of biomass. Certain chemicals measured with potentially high inhalation toxicity have been shown to be major components of biomass combustion including phenol [61], 2-methoxyphenol [59, 60], and o-cresol [63]. In our study, 2-methoxyphenol was measured with the most abundant signal (by an order of magnitude) of all chemicals detected in biomass combustion emissions with inhalation toxicity (categorized as high inhalation toxicity). Methoxyphenols, of which 2-methoxyphenol is one of the most abundant, are derived from lignin during combustion and have been shown previously to be the main combustion products of biomass burning for a number of wood materials [59, 60] Cresols and chlorinated organic species were some of those with both high rat LD50s and very high inhalation toxicity. Cresols can cause skin irritation for hypersensitive individuals and a maximum level of 5 ppm is recommended in air [67, 68]. Four chlorinated species were also detected, with hexachlorobenzene being the most abundant by orders of magnitude, which corresponds to literature pointing to biomass burning being a major source of this chemical with high bioaccumulation, persistence, and potential toxicity [58, 69]. Of the other species not commonly reported as biomass products they may either be false positives, trace species, or components not yet thoroughly investigated. Therefore, it would be of importance to look further into these chemicals, as well as the chemicals previously discussed, to understand their role in human health.

Increased cytotoxicity of the chronic bronchitis-like bronchial model (bro-ALI-CB) to wood smoke compared to normal bronchial (bro-ALI) and alveolar models (alv-ALI) reflect higher susceptibility of the predisposed condition. The alveolar model exhibited slightly higher cytotoxicity compared to the normal bronchial model when exposed to wood smoke. Increased oxidative stress due to wood smoke exposure in bro-ALI and bro-ALI-CB but not alv-ALI was detected by elevated cellular total ROS and NFkB levels. Wood smoke extract induced oxidative stress in the lung have been reported by other studies as well [17–25].

The enrichment of canonical pathway terms (Fig. 10) such as production of nitric oxide and ROS, apoptosis signaling, NRF2-mediated oxidative stress response, NFkB activation, P13/AKT, metabolism of ROS, transient infantile mitochondrial myopathy, oxidative phosphorylation, xenobiotic metabolism of aryl hydrocarbon receptor signaling pathway, aryl hydrocarbon receptor signaling, and mitochondrial dysfunction in alv-ALI reflects the adverse oxidative effects of wood smoke even though total ROS was not increased. It is important to consider the intrinsic different physiological characteristics of the bro-ALI, bro-ALI-CB, and alv-ALI models that may influence the pattern and time of response to wood smoke exposure. Moreover, the normal bronchial and chronic bronchitis-like bronchial models are developed from human primary bronchial epithelial cells whereas the alveolar model is developed from immortalized lung adenocarcinoma cell line exhibiting characteristics of type II pneumocytes. Further, the bronchial model differentiates into Club cells, goblet cells, basal cells, ciliated cells etc. and each have their unique cellular and molecular characteristics. The chronic bronchitis-like bronchial model contains more mucous producing cells and more mucous. On the other hand, presence of tight junction protein 1, lamellar bodies, surfactant protein C, microvilli, lipid bodies, desmosome, and tight junctions are features of the alveolar model. The role as a protective physical barrier of the surfactant layer of the alveoli has also been demonstrated in cell culture [70]. Similar observations were also made in other exposure studies by us as well [34, 36].

It has been demonstrated that sub-chronic exposure to cigarette smoke and electronic cigarette aerosol disrupts barrier function in human bronchial epithelial cells cultured at ALI [71]. TEER value of both cigarette smoke and electronic cigarette exposed lung models were reduced by half compared to air control [71]. Correspondingly, transcript levels of E cadherin were also significantly (nearly 40%) reduced [71]. However, in response to wood smoke exposure TEER values were not altered in any of the three models in this study. Consistent with that, expression of *CLDN3, CDHR3,* and *CDHR4* as tight junction genes was increased in the wood smoke exposed normal bronchial model. Expression of only *PCDHGA5* as a tight junction gene increased in the wood smoke exposed chronic bronchitis-like bronchial model. Increased expression of some of the tight junction related genes following wood smoke exposure observed in this study may reflect protective function since barrier integrity was intact. However, it remains to be investigated if longer duration of wood smoke exposure alters barrier function.

Although somewhat surprising, we did not detect any altered levels of the pro-inflammatory cytokines in the alv-ALI models exposed to wood smoke. In contrast, reduced levels of IL6 and TNFα were detected between 48 and 72 h since the start of exposure in bro-ALI and bro-ALI-CB. The reduction was prolonged and slightly more pronounced in the chronic bronchitis-like bronchial model. A dose gradient response of the cytokine release plausibly at even earlier and later stages may reveal the actual response pattern which was beyond the scope of this study. The reduced levels of pro-inflammatory cytokines may be considered as an indication of suppressed inflammatory response via. the aryl hydrocarbon receptor pathway as reported in case of cigarette smoke [72–74]. Nonetheless, enrichment of the terms acute phase response signaling, hypercytokinemia/hyperchemokinemia, cellular infiltration by granulocytes, immune mediated inflammatory diseases, and inflammation of respiratory system component in all the three models indicate common mechanisms of proinflammatory response (Fig. 10). Similarly, enrichment of terms IL10-, IL17-, TNFR1-, IL1-, IL17F-, IL13-signaling, pulmonary fibrosis idiopathic signaling pathway, chronic respiratory disorder, inflammation of lung, and chronic respiratory disorder in either single or two overlapping models reflects the underlying inflammatory processes triggered due to wood smoke exposure. Other studies with wood smoke extract or wood smoke particles also reported inflammation as an adverse outcome pathway in the lung [17–25]. Most of these pathways are also reported in relation to ambient particulate matter exposure as well [75]. Household air pollution due to biomass burning in low-middle income countries is estimated to contribute to at least 30% of ambient particulate matter and therefore observation of common enriched pathways is expected [76].

Mucins are the primary solid layer of the airway mucus that serves as the first line of defense against inhaled toxicants in the respiratory tract [77]. Mucins are produced from mucous secreting cells located in the bronchial epithelia [77]. In the lungs, mucous is responsible for innate airway defense and airway surface hydration. MUC5AC and MUC5B glycoproteins are the primary solid components of the lung mucous layer [77]. Other mucins are also present in the lungs. Mucous hypersecretion is commonly associated with several respiratory disease conditions such as asthma, chronic bronchitis, COPD, and cystic fibrosis [77]. Wood smoke exposure resulted in increased expression of *MUC1*, *MUC5AC*, and *MUC5B* in the chronic bronchitis-like bronchial model. In the case of normal bronchial model, expression of only *MUC12* was increased following wood smoke exposure. Differential regulation of *MUC1* and *MUC12* in the lung has also been associated with non-small-cell lung cancer [78]. The findings indicate that wood smoke exposure can affect mucin homeostasis particularly in the chronic bronchitis-like bronchial model. Further, the findings are in line with a previous study reporting long-term exposure of biomass smoke as a risk factor for chronic bronchitis [56].

One of the most interesting findings of the pathway analysis was the enrichment of the terms primary ciliary dyskinesia, syndromic ciliopathy, movement of cilia, and Kartagener syndrome in the normal- and chronic bronchitis-like bronchial mucosa models in response to wood smoke exposure. In the airways, cilia functions in concert with airway mucus for mucocililary clearance to remove inhaled particles and pathogens [79]. The findings suggest that long-term biomass smoke exposure may lead to dysfunction of cilia leading to disruption of the healthy respiratory epithelium and increased susceptibility to acquired lung disorders. The enrichment was more pronounced in the normal bronchial model consistent with the differential regulation of 187 genes (up-regulated: 180; down-regulated: 7) genes related to the gene ontology term ‘cilium’ and ‘motile cilia’. This is particularly relevant considering the recent observation of greater bronchial involvement in biomass smoke induced COPD than tobacco smoke induced COPD [11, 14–16]. There are ongoing discussions regarding inclusion of ciliopathies and ciliopathy-associated COPD as a COPD endotype [80].

Primary ciliary dyskinesia results in impairment of ciliary function and defective mucociliary clearance in the respiratory tract. Patients with primary ciliary dyskinesia are prone to frequent and serious respiratory infections leading to progressive destruction of the lung architecture [79, 81]. Primary ciliary dyskinesia arises from defects within genes involved in the transport, assembly, and function of motile cilia [82]. Motile cilia are present on a group of specialized epithelial surfaces and act as a mucociliary escalator moving fluid and debris in a synchronized and unidirectional way at the air–liquid interface [82]. The primary cilia on the other hand are single non-motile organelles that are present in almost all cells of the human body and act as sensors for physical and chemical stimuli and are also responsible for organogenesis and normal development [82]. Dysfunction of non-motile cilia leads to syndromic disorders [82]. Evidence supporting the sensory role of motile cilia has also emerged [82]. Defects in certain ciliary proteins important for both primary (non-motile) and motile cilia can lead to ciliopathic phenotypes with overlapping clinical features [82]. Acquired disorders of airway cilia due to structural and/ or functional abnormalities because of impaired mucociliary clearance arising from cigarette smoke exposure and environmental pollutants have been reported. Nasal biopsies from Mexico City residents exposed to high levels of ambient air pollution showed patches of short cilia and regions of cilia loss [79]. Exposure of cultured human bronchial epithelial cells to diesel exhaust particles exhibited reduced ciliary beating. Several compounds present in indoor air pollution (such as formaldehyde, acrolein, ammonia) affect the ciliary beating, ciliary structure, and mucus flow [79]. However, in this study we have not performed any assessment of the ciliary structure and function.

To outline some of the study limitations, a continuous monitoring system for at least some of the chemicals, CO, and particle number concentrations during the actual exposure would have been advantageous. Further, if the exposure experiments could have been performed with primary bronchial epithelial cells collected from multiple healthy donors, donors with chronic bronchitis, and donors with primary ciliary dyskinesia would have made the study more clinically relevant. Further, multiple time point analysis of toxicological endpoints may provide more accurate information on the pattern of responses in each lung mucosa model. As understandable, incorporation of all the features in a single study is challenging.

## Conclusions

To conclude, a standardized and direct wood smoke exposure resulted in increased ROS and NFkB in the normal- and chronic bronchitis-like bronchial mucosa models but not in the alveolar model indicating differential oxidative stress response pattern in different regions of the respiratory tree. However, oxidative stress response as detected through transcriptomic analysis indicates wood smoke induced oxidative stress and pro-inflammatory response in normal- and chronic bronchitis-like bronchial models, as well as in the alveolar model. Barrier integrity was unaffected in all the models for the exposure regimen used in this study. Indication of mucin homeostasis being affected was observed in the chronic bronchitis-like model following wood smoke exposure. Patterns of secretion of proinflammatory cytokines were also different in different models following wood smoke exposure. Taken together, these observations warrant multi-time point based analysis in such exposure studies. The enrichment of the pathways primary ciliary dyskinesia, syndromic ciliopathy, and movement of cilia in the wood smoke exposed normal- and chronic bronchitis-like bronchial mucosa models is in line with the reports on greater bronchial involvement in case of biomass smoke induced COPD compared to tobacco smoke induced COPD. Thus, in future more emphasis is needed on understanding household air pollution mediated respiratory ciliopathy. Further, it needs to be investigated how the toxicity alters with different wood types and solid fuel used in low-middle income countries for domestic purposes. Hence, personal exposure assessments along with health risk assessments are required for field studies.

### Supplementary Information

**Additional file 1.** Supplementary Figures and Tables.

## Data Availability

All data presented in the study are available on request from the corresponding authors and are also included in the supplementary section. RNA sequencing data is deposited at the Gene Expression Omnibus database at NCBI [GSE236857; https://www.ncbi.nlm.nih.gov/geo/; accessed on 17th January 2024].

## Abbreviations

ALI: Air–liquid interface
alv: Alveolar
alv-ALI: Alveolar model
bro: Normal bronchial
bro-ALI: Normal bronchial mucosa model
bro-ALI-CB: Chronic bronchitis-like bronchial model
CB: Chronic bronchitis-like bronchial
CO: Carbon monoxide,
CO_2_: Carbon dioxide
COPD: Chronic obstructive pulmonary disease
EI: Electron impact
ELISA: Enzyme linked immune sorbent assays
GC: Gas chromatography
GC-HRMS: Gas chromatography high resolution mass spectrometer
h: Hour
HAP: Household air pollution
IL: Interleukin
IQR: Interquartile ranges
LDH: Lactate dehydrogenase
MFI: Mean fluorescent intensity
min: Minutes
NFkB: Nuclear factor kappa-light-chain-enhancer of activated B cells
PAHs: Polycyclic aromatic hydrocarbons
PBEC: Primary bronchial epithelial cells
PI: Propidium iodide
PM: Particulate matter
qRT-PCR: Quantitative real time polymerase chain reaction
RNAseq: RNA sequencing
ROS: Reactive oxygen species
SOD3: Superoxide dismutase 3, extracellular
TNF: Tumor necrosis factor
TEER: Transepithelial electrical resistance
VOC: Volatile organic compounds
