# Supplementary material for: Assessment of wood smoke induced pulmonary toxicity in normal- and chronic bronchitis-like bronchial and alveolar lung mucosa models at air–liquid interface
Source: Respir Res. 2024 Jan 20;25:49. doi: 10.1186/s12931-024-02686-5 (PMC10799428; doi:10.1186/s12931-024-02686-5)

**Supplementary Material**

**Assessment of wood smoke induced pulmonary toxicity in normal- and chronic bronchitis-like bronchial and alveolar lung mucosa models at air-liquid interface.**

Swapna Upadhyay^1*^, Mizanur Rahman^1^, Selina Rinaldi^1^, Jeremy Koelmel^2^, Elizabeth Z. Lin^2^, Padukudru Anand Mahesh^3^, Johannes Beckers^4,5,6^, Gunnar Johanson^1^, Krystal J. Godri Pollitt^2^, Lena Palmberg^1^, Martin Irmler^4^, Koustav Ganguly^1*^.

^1^Unit of Integrative Toxicology, Institute of Environmental Medicine (IMM), Karolinska Institutet, Stockholm, Sweden.

^2^Department of Environmental Health Sciences, Yale School of Public Health, Yale University, New Haven, CT, US.

^3^Department of Respiratory Medicine, JSS Medical College, JSS Academy of Higher Education and Research, Mysuru 570015, India.

^4^Institute of Experimental Genetics, Helmholtz Zentrum München, Deutsches Forschungszentrum für Gesundheit und Umwelt (GmbH), 85764, Neuherberg, Germany

^5^German Center for Diabetes Research (DZD e.V.), 85764 Neuherberg, Germany

^6^Chair of Experimental Genetics, Technical University of Munich, 85354 Freising, Germany

| Swapna Upadhyay | swapna.upadhyay@ki.se |
| --- | --- |
| Mizanur Rahman | mizanur.rahman@ki.se |
| Selina Rinaldi | Selina.Rinaldi@bfr.bund.de |
| Jeremy Koelmel | jeremykoelmel@gmail.com |
| Elizabeth Z. Lin | elizabeth.lin@yale.edu |
| Padukudru Anand Mahesh | pamahesh@jssuni.edu.in |
| Johannes Beckers | Johannes.beckers@helmholtz-munich.de |
| Gunnar Johanson | gunnar.johanson@ki.se |
| Krystal J. Godri Pollitt | krystal.pollitt@yale.edu |
| Lena Palmberg | lena.palmberg@ki.se |
| Martin Irmler | martin.irmler@helmholtz-munich.de |
| Koustav Ganguly | koustav.ganguly@ki.se |

**^*^Correspondence**

Koustav Ganguly (koustav.ganguly@ki.se)

Unit of Integrative Toxicology, Institute of Environmental Medicine, Karolinska Institutet, Stockholm, 171 77, Sweden

Swapna Upadhyay (swapna.upadhyay@ki.se)

Unit of Integrative Toxicology, Institute of Environmental Medicine, Karolinska Institutet, Stockholm, 171 77, Sweden

**Financial support:** Swedish Heart Lung Foundation, The Swedish Foundation for International Cooperation in Research and Higher Education (STINT), Swedish Research Council, and Forska Utan Djurförsök/ Do Research without animal.

**Legends Supplementary tables**

**Supplementary table ST1 – ST7 are provided in the EXCEL file** (Supplementary Tables ST1-7)

**Supplementary table ST1:** List of regulated genes (total: 1262; p ≤ 0.01 compared to sham) in the bro-ALI model following wood smoke exposure. 817 genes were up-regulated and 445 were down-regulated. bro-ALI: normal bronchial mucosa model at air-liquid interface

**Supplementary table ST2:** Shown are 56 significantly enriched canonical pathways (Fisher`s Exact test p-value < 0.05) associated with the set of 1262 regulated genes in the bro- ALI model following wood smoke exposure compared to sham. z-scores > 2 indicate activation, z-scores < -2 indicate inhibition. bro-ALI: normal bronchial mucosa model at air-liquid interface.

**Supplementary table ST3:** List of regulated genes (total: 329; p ≤ 0.01 compared to sham) in the bro-ALI-CB model following wood smoke exposure. 141 genes were up-regulated and 188 were down-regulated. bro-ALI: normal bronchial mucosa model at air-liquid interface; bro-ALI-CB: chronic bronchitis-like bronchial mucosa model.

**Supplementary table ST4:** Shown are 36 significantly enriched canonical pathways (Fisher`s Exact test p-value < 0.05) associated with the set of 329 regulated genes in the bro- ALI-CB model following wood smoke exposure compared to sham. z-scores > 2 indicate activation, z-scores < -2 indicate inhibition. bro-ALI: normal bronchial mucosa model at air-liquid interface; bro-ALI-CB: chronic bronchitis-like bronchial mucosa model.

**Supplementary table ST5:** List of regulated genes (p ≤ 0.01 compared to sham) in the alv-ALI model following wood smoke exposure. 33 genes were up-regulated and 69 were down-regulated (total: 102). alv-ALI: alveolar mucosa model at air-liquid interface.

**Supplementary table ST6:** Shown are 108 significantly enriched canonical pathways (Fisher`s Exact test p-value < 0.05) associated with the set of 102 regulated genes in the alv-ALI model following wood smoke exposure compared to sham. z-scores > 2 indicate activation, z-scores < -2 indicate inhibition. alv-ALI: alveolar mucosa model at air-liquid interface.

**Supplementary table ST7:** Genes associated with the gene ontology terms ´cilium`(GO:0005929) or ´motile cilium`( GO:0031514 ) in mouse and regulated in either analysis: 187 genes (180 up-regulated, 7 down-regulated) in a bro-ALI model exposed to wood smoke compared to sham ( p ≤ 0.01) or 26 genes (25 up-regulated, 1 down-regulated) in a bro-ALI-CB model exposed to wood smoke compared to sham (p ≤ 0.01). bro-ALI: normal bronchial mucosa model at air-liquid interface; bro-ALI-CB: chronic bronchitis-like bronchial mucosa model.

**Supplementary table ST8:** Expression of selected genes assessed by quantitative real time PCR (qRT-PCR).

**Supplementary Information-B:** Wood smoke composition in EXCEL file.

**Supplementary table ST8:** Expression of selected genes (based on significant differential regulation in RNAsequencing data) were assessed by quantitative real time PCR (qRT-PCR) in wood smoke exposed bro-ALI and bro-ALI-CB compared to the corresponding sham (n = 6 independent experiments per exposure condition). None of the selected genes were significantly altered in the alv-ALI model (RNAseq data) and therefore qRT-PCR was not carried out for the alveolar model. qRT-PCR data are expressed as fold changes (median; non-parametric Wilcoxon signed rank test, significance p<0.05) and *ACTB* is used as the reference gene control. Pre-designed primer pairs for all genes to perform qRT-PCR were obtained from Sigma Adrich, Amsterdam, Netherlands (<https://www.sigmaaldrich.com/SE/en/semi-configurators/kicqstartPrimers>). RNAseq data are available in the supplementary tables ST1 (bro-ALI), ST3 (bro-ALI-CB), and ST5 (alv-ALI). **↑:** up-regulated**; ↓:** down-regulated **(-); ALI**: air-liquid interface; **alv-AL**I: alveolar mucosa model at ALI; **bro-ALI**: normal bronchial mucosa model at ALI; **bro-ALI-CB**: chronic bronchitis-like bronchial lung mucosa model; **NC**: no change.

| **Gene symbol** | **Gene name** | **Ensembl gene ID** | **bro-ALI** | | **bro-ALI-CB** | | **Alv-ALI** |
| --- | --- | --- | --- | --- | --- | --- | --- |
|  |  |  | **RNAseq** | **qRT-PCR** | **RNAseq)** | **qRT-PCR** | **RNAseq** |
|  |  |  | **Fold change** | **Fold change** | **Fold change** | **Fold change** | **Fold change** |
| **Barrier Function** | | | | | | | |
| ***TJP1*** | Tight junction protein 1 | ENSG00000104067 | NC | NC | -1.28 **↓** | NC | NC |
| ***TJP3*** | Tight junction protein 3 | ENSG00000105289 | 1.46 **↑** | NC | NC | NC | NC |
| ***CLDN1*** | Claudin 1 | ENSG00000163347 | -1.50 **↓** | NC | NC | NC | NC |
| ***CLDN3*** | Claudin 3 | ENSG00000165215 | 3.05 **↑** | 2.95 **↑** | NC | NC | NC |
| ***CLDN4*** | Claudin 4 | ENSG00000189143 | 1.40 **↑** | NC | NC | NC | NC |
| ***CDHR3*** | Cadherin related family member 3 | ENSG00000128536 | 2.92 **↑** | 2.24 **↑** | NC | NC | NC |
| ***CDHR4*** | Cadherin related family member 4 | ENSG00000187492 | 9.59 **↑** | 6.81 **↑** | NC | NC | NC |
| ***PCDHGA5*** | Protocadherin gamma subfamily A, 5 | ENSG00000253485 | NC | NC | 5.59  **↑** | 6.75 **↑** | NC |
| **Mucin** | | | | | | | |
| ***MUC1*** | Mucin, cell surface associated 1 | ENSG00000185499 | -1.44 **↓** | NC | 1.22  **↑** | 1.52 **↑** | NC |
| ***MUC5AC*** | Mucin, oligomeric mucus/gel-forming 5AC | ENSG00000215182 | NC | NC | 1.83  **↑** | 2.47  **↑** | NC |
| ***MUC5B*** | Mucin, oligomeric mucus/gel-forming 5B | ENSG00000117983 | NC | NC | 1.47 **↑** | 4.84 **↑** | NC |
| ***MUC12*** | Mucin, cell surface associated 12 | ENSG00000205277 | 3.99 **↑** | 3.45 **↑** | NC | NC | NC |
| ***MUC16*** | Mucin, cell surface associated 16 | ENSG00000181143 | NC | NC | 1.40  **↑** | NC | NC |

**Legends Supplementary figures**

**Supplementary figure SF1:** Pilot studies were performed by exposing normal bronchial mucosa models cultured at air-liquid interface (bro-ALI) to wood smoke (1:20 diluted with clean filtered air) for 5, 10, and 15 minutes to determine the duration of exposure. (n = 1; 3 technical replicates).

**a.** Lactate dehydrogenase (LDH) assay was used to assess the cytotoxicity of the 3 exposure durations, and none were found to be cytotoxic. Positive control: kit provided; Negative control: culture media before exposure (before). Transcript levels of (**b)** superoxide dismutase 3, extracellular (*SOD3*) and (**c**) tumor necrosis factor (*TNF*) was assessed as markers of oxidative stress and pro-inflammatory response respectively. Based on the observations of the pilot study, the medium exposure duration of 10 minutes was used for performing repeated exposure studies.

**a.**


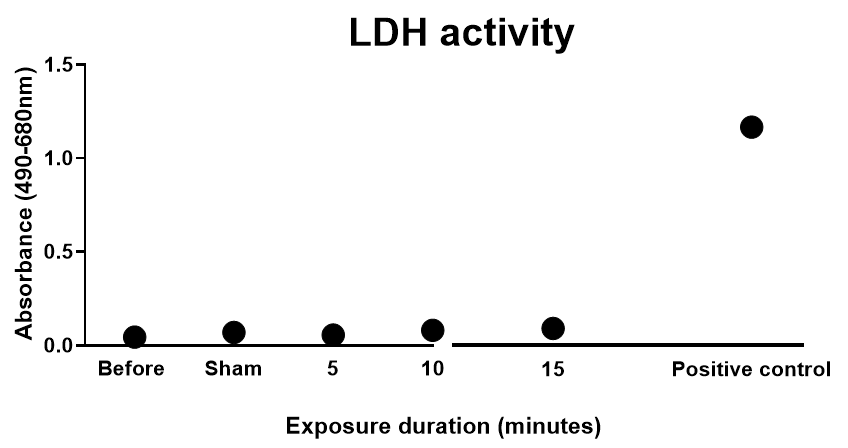


**b.**


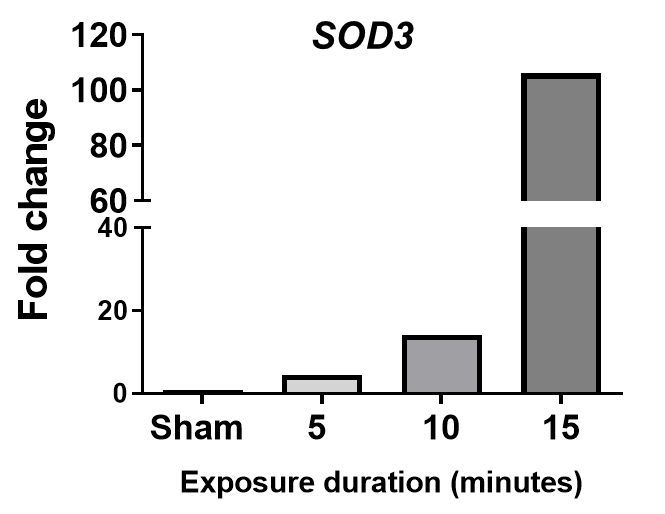


**c.**


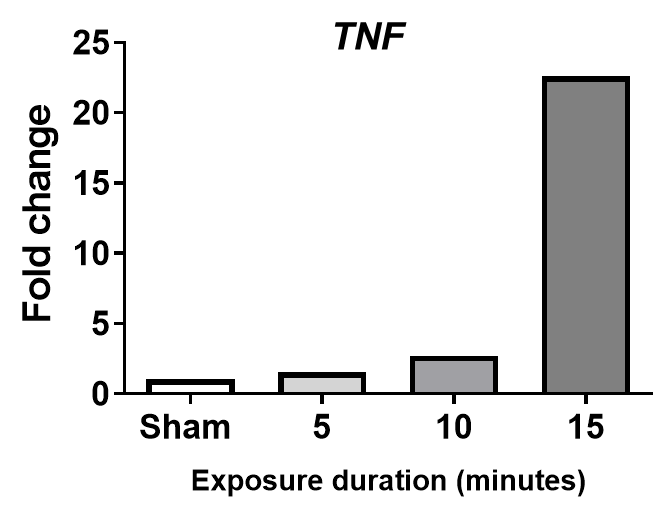


**Supplementary figure SF2:** Particle size distribution of wood smoke generated using a temperature controlled electric smoker set at 80°C and pre-weighed 30g of smoking shavings-hickory. The particle size distribution ranged between 0.25 - 2.5 µm with the three peak mode diameters around 0.25, 0.58, and 0.28 µm in descending order. (Data are represented as mean ± SEM of n = 8 measurements). Particle number concentration is used as the instrument output (counts) and shown as counts per cm^3^.


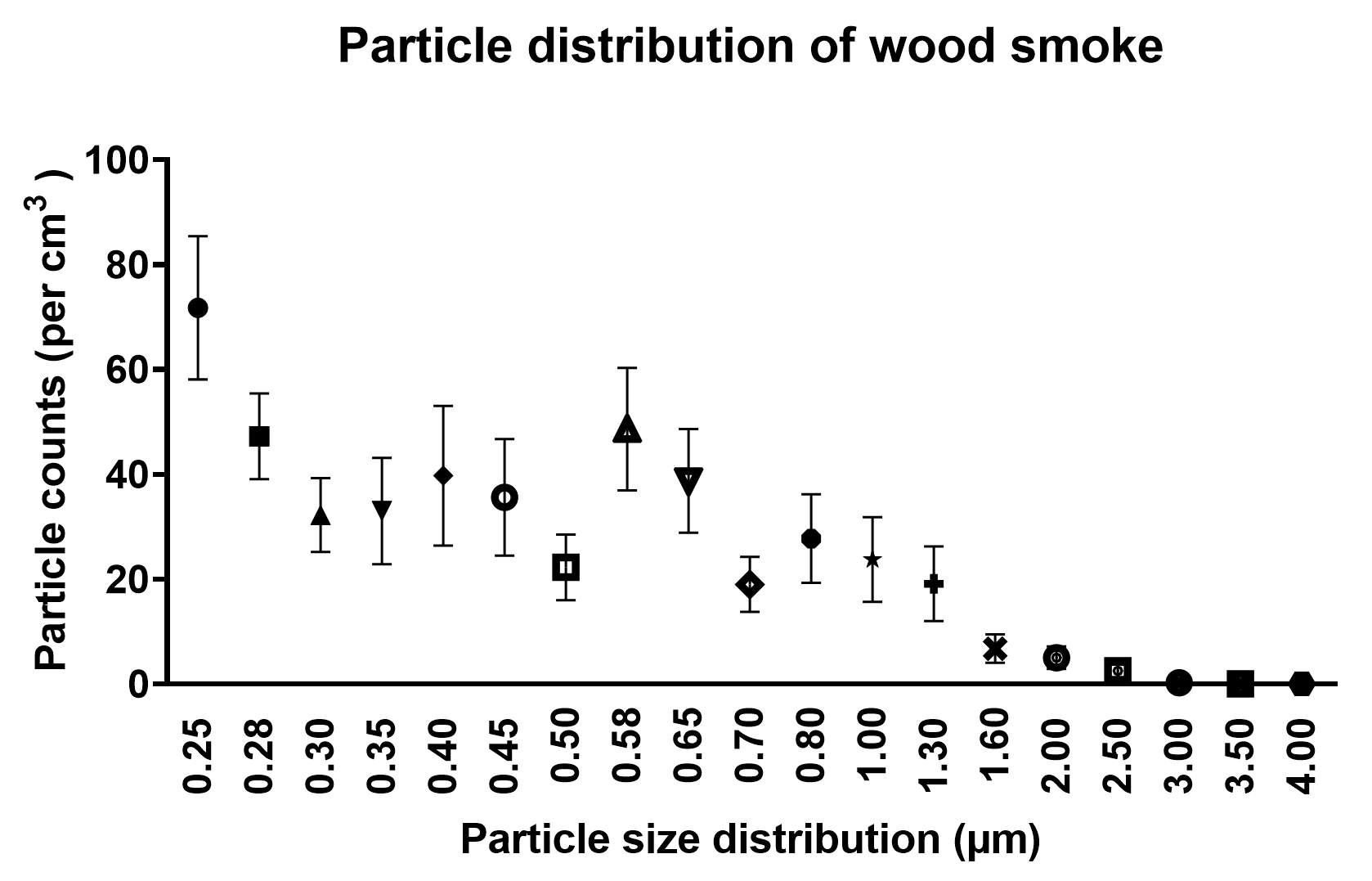


**Supplementary figure SF3:** Assessment of cytotoxicity using (**a, b, c**) membrane integrity based propidium iodide (PI) staining by flow cytometry in sham exposed and wood smoke exposed bro-ALI, bro-ALI-CB, and alv-ALI models. Data are shown as percentages of positive PI cells and interquartile ranges; n = 3 independent experiments per exposure condition; non-parametric statistical analysis (Wilcoxon signed rank test), *p< 0.05.

ALI: air-liquid interface; alv-ALI: alveolar mucosa model at ALI; bro-ALI: normal bronchial mucosa model at ALI; bro-ALI-CB: chronic bronchitis-like bronchial lung mucosa model.


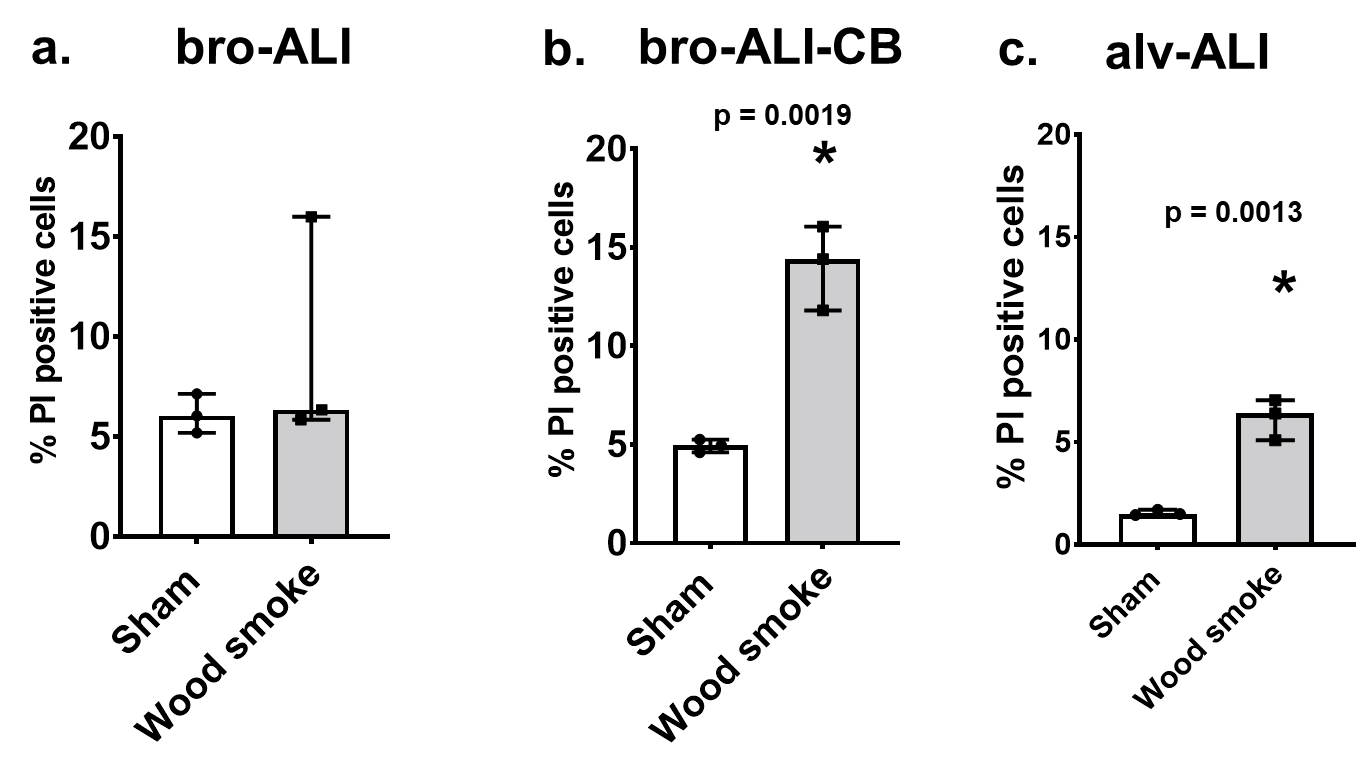

Supplement: Supplementary file 1 — Additional file 1. Supplementary Figures and Tables. [file 12931_2024_2686_MOESM1_ESM.zip › 02-Suppl Fig SF1-SF3 suppl tab ST1-ST8-Final 20-12-2023_ESM.docx]
